# A Modular T7 RNA Polymerase Toolbox Linking Selective miRNA Detection to Signal Amplification and Protein Expression

**DOI:** 10.1021/jacs.5c16214

**Published:** 2025-11-19

**Authors:** Maria Vonk-de Roy, Andreas Walther

**Affiliations:** Life-Like Materials and Systems, University of Mainz, Duesbergweg 10-14, 55128 Mainz, Germany

## Abstract

Cells process and integrate diverse molecular signals through complex biochemical reaction networks combining sensing, computation, and response. Synthetic analogs in the form of chemical reaction networks (CRNs) still fall short in parallel and selective processing and often suffer from limited modularity, incompatibilities, or leakage. Here, we present a versatile and leakage-free T7 RNA polymerase (T7 RNAP) toolbox that bridges from selective oligonucleotide detection to gated transcription and finally protein expression in a programmable manner. Central to our system is the design of modular T7-Locks, that release active T7 promoters only upon specific oligonucleotide binding to trigger transcription. This enables quantitative and orthogonal miRNA detection with ON/OFF ratios exceeding 100. We further demonstrate that incorporation of site-specific transcription terminators via C12 spacers inside T7 templates enables controlled signal amplification through positive feedback. Finally, we establish miRNA-triggered protein expression in a cell-free TX-TL system using specifically engineered sticky-end genes that are gated through T7-Locks on a transcriptional level. This modular platform connects input sensing, amplification, and output synthesis, providing a generalizable strategy for DNA or RNA-driven information processing in synthetic biology.

## Graphical abstract

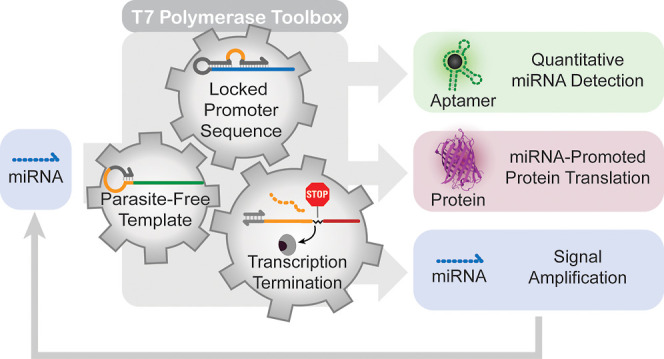

## Introduction

Cells contain highly complex (bio)­chemical reaction networks (CRNs) that enable sophisticated information processing with high tunability.^1^ Among other functions, CRNs facilitate the ability of cells to sense molecular signals and convert them into functional outcomes such as gene expression. Positive feedback can enhance activity, and biological CRNs can deal with noise and fluctuation through redundancies to prevent leakage reactions from reaching unwanted systemic effects.^2,3^ In essence, high CRN complexity renders the cell a highly efficient multiplexing molecular computing system. In contrast, in vitro applications of CRNs, such as for biosensing, molecular computing, or for the design of life-like systems rather benefit from streamlined strategies of ideally low complexity and with low energetic or component footprint.^4–7^

DNA and RNA offer unique programmability, making them a robust foundation for many engineered synthetic CRNs, such as those built on strand displacement reactions (SDRs), the polymerase–exonuclease–nickase (PEN) toolbox, or ATP-driven circuits.^8–14^ Orthogonal nucleic acid interactions and predictable SDRs are exploited in these systems for molecular sensing, logic computation, and controlled gene expression.^15–19^ Additionally, their inherent compatibility with enzymes provides extra layers of control, enabling, for example, nonlinear signal amplification and oscillatory circuits.^20–22^ However, synthetic CRNs with desirable functions often come with system-specific limitations—particularly in transducing low information inputs to high information outputs. A notable example of this would be the sensing of short microRNAs (miRNAs) and their transduction into arbitrary RNA outputs or even proteins, which would constitute a highly valuable biosensor.

Here, we present a modular T7 RNA polymerase (T7 RNAP)-based toolbox as a CRN element for programmable transcription regulation, emphasizing input/output modularity and leakage-free design (i.e., low parasitism). T7 RNAP is a robust enzyme that catalyzes the synthesis of RNAs starting from a defined region on a DNA template—the T7 promoter sequence.^23^ In combination with controlled SDRs, switches, and RNA-degrading enzymes, it forms the basis of versatile CRNs, for instance so-called genelets, that can emulate complex behaviors.^20,22,24–30^ Major drawbacks include limited flexibility of nucleic acid–based inputs to trigger transcription, as activation requires completion of the constrained T7 promoter sequence. This restriction limits the potential for biosensing and universal signal processing within T7 RNAP-based CRNs. Additionally, extending the output beyond RNA to the protein level would be desirable to achieve higher-level output functions in complex systems. To address these challenges, we first design a T7-Lock system that enables oligonucleotides of diverse classes and sequences to serve as universal signal inputs to trigger transcription. By incorporating a fluorescent readout, we apply this system to develop a method for both quantitative and qualitative detection of miRNAs. Next, we introduce a novel tool for the site-specific termination of T7 transcription, which we leverage to generate a positive feedback loop for signal amplification. Finally, we demonstrate the versatility of the toolbox by integrating it into a cell-free transcription-translation (TX-TL) environment, achieving miRNA-induced protein synthesis as an example of a powerful high-information output.

## Results and Discussion

### General Design

Figure 1 summarizes our approach. The T7 RNAP toolbox builds on the well-established principle of T7 RNAP-mediated transcription from a T7 DNA template, in the presence of its T7 promoter.^23^ At the core of our design is the so-called T7-Lock. The T7-Lock structure is engineered to sequester the T7 promoter—the essential element required for initiating transcription, which can only be revealed by binding to a specific (mi)­RNA or DNA key (Figure 1a). In addition, we suppress parasitic transcription leakage by folding of the T7 promoter binding site into a hairpin. Once the T7-Lock is opened, the T7 promoter can hybridize to T7 DNA templates by opening the hairpin and initiate transcription (Figure 1b). This endows the system with universal sensing capability to diverse miRNA inputs, and flexible coding of arbitrary RNA outputs as encoded in the T7 template (Figure 1c). We will further show how the T7 template design can be modified to enable autocatalysis or even be coupled to protein synthesis in a cell-free TX-TL system. The latter significantly expands the synthetic biology toolbox to trigger protein synthesis in response to small miRNA inputs—a process not found in natural biological systems, where miRNAs typically suppress translation through processes such as controlled mRNA degradation.^31^

**Figure 1:**
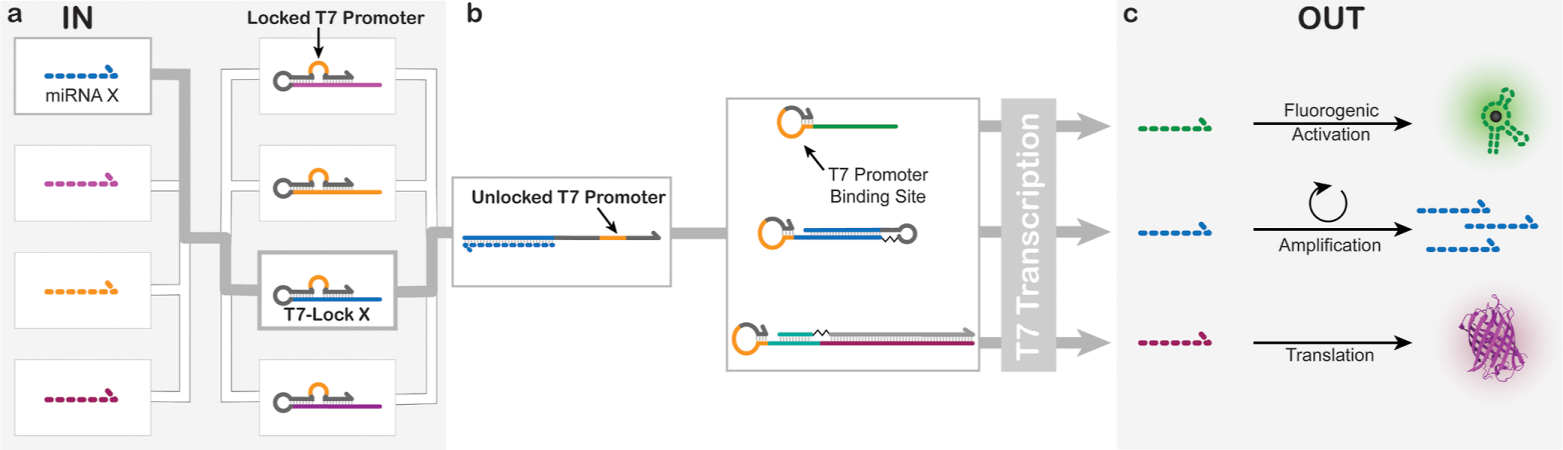
Main features of the T7 RNAP toolbox. (a) T7-Locks only expose their sequestered T7 promoter sequence upon activation by their corresponding miRNA key. (b) The unlocked T7 promoter is then available to bind to a wide range of templates containing a T7 promoter binding site, thereby initiating T7 transcription. (c) Depending on the template design, a wide range of RNA sequences can be transcribed. These outputs can subsequently be used for fluorescence readout, autocatalytic signal amplification, or protein translation.

### Reducing Leakage in T7 RNAP-Mediated Transcription by Folded T7 Templates

Figure 2a illustrates the essential DNA sequences for T7 RNAP-mediated transcription in more detail. The T7 promoter, a 17-nucleotide (nt) motif, contains a unique sequence that the T7 RNAP recognizes once it has hybridized to a DNA template containing its complementary sequence.^32,33^ Transcription is initiated in the region downstream of this site (Figure 2b). The transcribed region has no significant sequence constraints and allows for the free design of any RNA output. One convenient way to monitor transcription activity is the use of light-up aptamers, for which we selected the Spinach aptamer.^34^ The Spinach aptamer binds to 3,5-difluoro-4-hydroxybenzylidene imidazolinone (DFHBI), resulting in a GFP-like fluorescence signal, enabling real-time fluorescence measurements of the transcription rate and efficiency (Figure 2c). Figure 2d shows high ON/OFF ratios for transcription once the correct T7 promoter DNA sequence is added. In this classical system, using a linear template and direct promoter addition, a nonzero signal is present for a T7 promoter RNA or even a random, nonbinding DNA dummy input as well.

**Figure 2:**
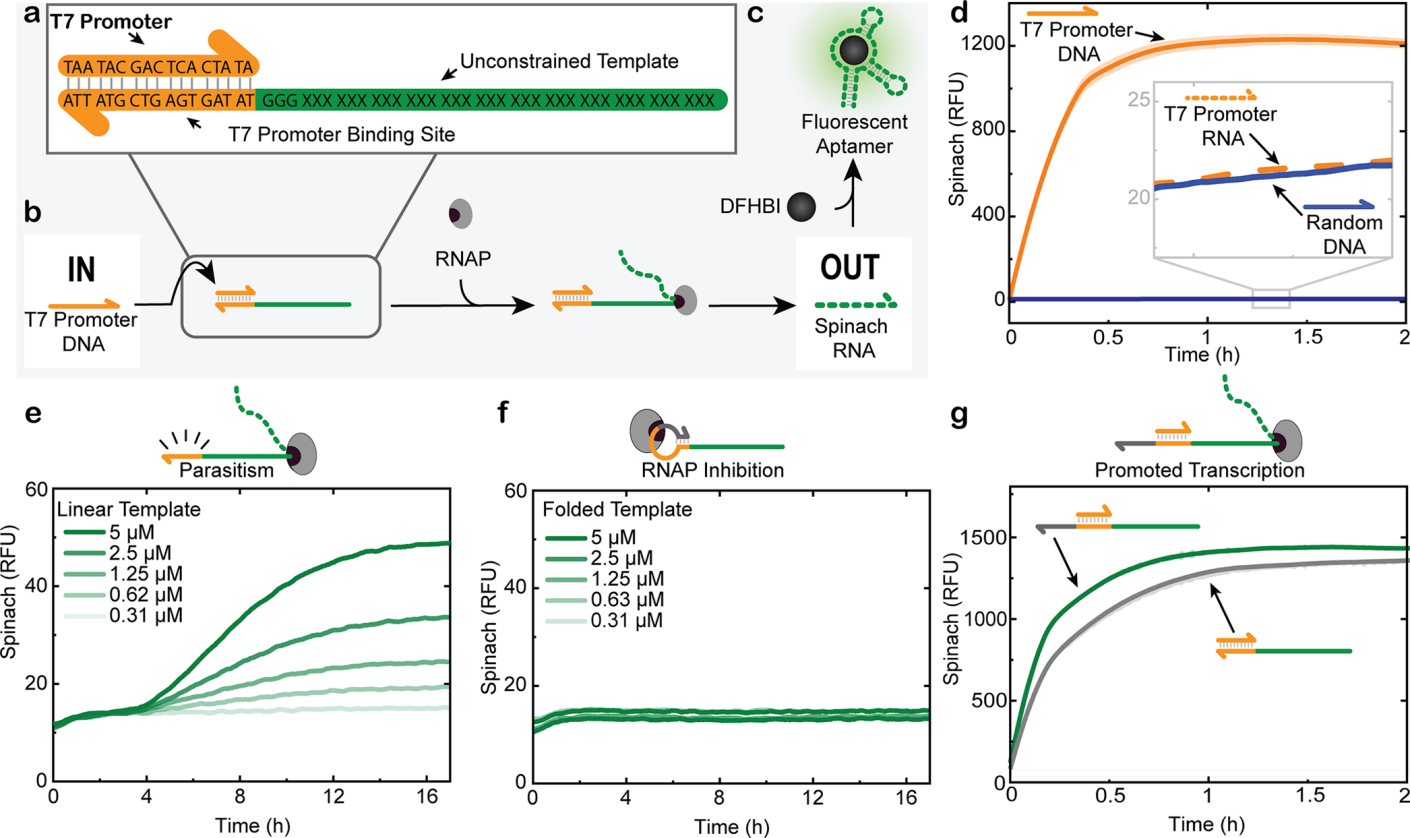
Activation, specificity, and suppression of parasitism of T7 RNAP-mediated transcription. (a) Schematics of a transcription template with a T7 promoter (orange), T7 promoter binding site (orange), and unconstrained region (green). (b) Schematic representation of transcription that is initiated upon hybridization of a DNA-based T7 promoter with the T7 promoter binding site on the template. No transcription occurs with an RNA-based T7 promoter or a random DNA strand. (c) The transcribed Spinach aptamer binds to DFHBI, emitting a fluorescent signal. (d) Real-time fluorescence measurements of DNA-based T7 promoter, RNA-based T7 promoter, and random DNA-triggered transcription of a template coding for the Spinach aptamer. Conditions: HiScribe T7 Quick High Yield RNA Synthesis Kit, 1.25 μM linear Spinach template, 40 mM HEPES, 125 mM KCl, 5 mM MgCl_2_, 62.5 mM NaCl, and 5 μM DFHBI at 37 °C. Input: 1.25 μM DNA-based T7 promoter, RNA-based T7 promoter, or random DNA (miDNA 141). All curves are averages of *n* = 5 measurements. Shaded areas (partly invisible as too small) represent the standard deviation. (e) Parasitic transcription observed in the absence of a T7 promoter, across a range of linear template concentrations. Conditions: HiScribe T7 Quick High Yield RNA Synthesis Kit, 0.31, 0.62, 1.25, 2.5, or 5 μM linear Spinach template, 40 mM HEPES, 125 mM KCl, 5 mM MgCl_2_, 62.5 mM NaCl, and 5 μM DFHBI at 37 °C. Input: none. Single measurements shown (f). Folded template design in absence of a T7 promoter fully suppresses parasitic transcription across the same template concentration range. Conditions: HiScribe T7 Quick High Yield RNA Synthesis Kit, 0.31, 0.62, 1.25, 2.5, or 5 μM folded Spinach template, 40 mM HEPES, 125 mM KCl, 5 mM MgCl_2_, 62.5 mM NaCl, and 5 μM DFHBI at 37 °C. Input: none. Single measurements shown. (g) Comparison of transcription activity for the folded versus the linear template after addition of the T7 promoter DNA. Conditions: HiScribe T7 Quick High Yield RNA Synthesis Kit, 1.25 μM linear or folded Spinach template, 40 mM HEPES, 125 mM KCl, 5 mM MgCl_2_, 62.5 mM NaCl, and 5 μM DFHBI at 37 °C. Input: 1.25 μM T7 promoter (*n* = 2; shaded areas (partly invisible as too small) represent the standard deviation).

In fact, one of the non-negligible issues of T7 RNAP-mediated transcription is the leakage or parasitism that occurs even in the absence of the promoter. Figure 2e shows that this background transcription correlates linearly with template concentration. Such unintended activity can compromise amplification strategies, such as for autocatalytic CRNs, that require strict dormancy. Obviously, partial recognition of the T7 promoter binding site by the T7 RNAP occurs even in the absence of the T7 promoter. To suppress this parasitic activity, we introduce a partially complementary sequence at the 3′ end of the T7 promoter binding site to fold it into a looped secondary structure (Figure 2f, more details in Figure S1). A comparison of a classical nonfolded and the folded T7 template demonstrates that this modification effectively suppresses background transcription (Figure 2e,f). The presence of the loop does not compromise the high ON/OFF ratio when the correct T7 promoter is added (Figure 2g). AlphaFold 3 simulations suggest that the folded template interacts with the T7 RNAP just as a linear template with a hybridized T7 promoter would (Figure S2).^35^ However, geometrical changes occur at the site of the intercalating loop of the T7 RNAP, where initiation of transcription occurs.^32^ This likely causes inhibition of the enzyme and effective suppression of parasitism. For our folded T7 templates, the RNA transcription output scales with the promoter concentration at constant template concentration (Figure S3). Delayed promoter addition after extended dormancy of up to 6 h does not impair the final RNA yield (Figure S4). Together, these findings validate a robust, leakage-free, triggerable transcription platform suitable for modular circuit design.

### Selective miRNA-Triggered T7 RNAP-Mediated Transcription Using T7-Locks

We next expand the T7 RNAP toolbox with the capacity for universal upstream sensing of diverse oligonucleotide inputs, where we focus on a selection of miRNAs. miRNAs are short noncoding RNAs of ∼19–24 nt in length and signatures of cell types and polarization, as well as indicators for diseases, rendering them highly relevant inputs for biosensing with CRNs.^36–40^ In humans alone, thousands of different miRNAs have been reported.^41,42^ Therefore, numerous miRNA detection methods have been reported, ranging from Northern blot to DNA SDRs with fluorescence read-out, RT-qPCR, and microarray technology.^37,43–46^ Previous T7 RNAP-based detection schemes however often required complex multistep pathways, suffering from significant parasitism, which limits the integration with more complex CRNs and raises the detection threshold.^47–50^ Higher sensitivity required integration with additional enzymes, e.g., Klenow DNA polymerase, T4 ligase, or reverse transcriptase.^51–54^

Since the T7 promoter sequence is constrained, miRNAs cannot be direct triggers for transcription. Therefore, we introduce the concept of T7-Locks, which sequester the T7 promoter sequence and prevent it from hybridizing with the promoter binding site on the template, and thus from inducing transcription (Figure 3a,b). More specifically, we designed the T7-Lock in a way that the T7 promoter sequence is confined in a loop and flanked by adjacent sequences that can be flexibly chosen, allowing full customization for diverse inputs. Additionally, a toehold region is present to unlock the T7-Lock using a key strand complementary to the blue part, thereby releasing the free T7 promoter sequence that is now able to bind to the template and induce transcription. The entire T7-Lock is in a hairpin configuration to ensure high effective molarity and tight binding.

**Figure 3:**
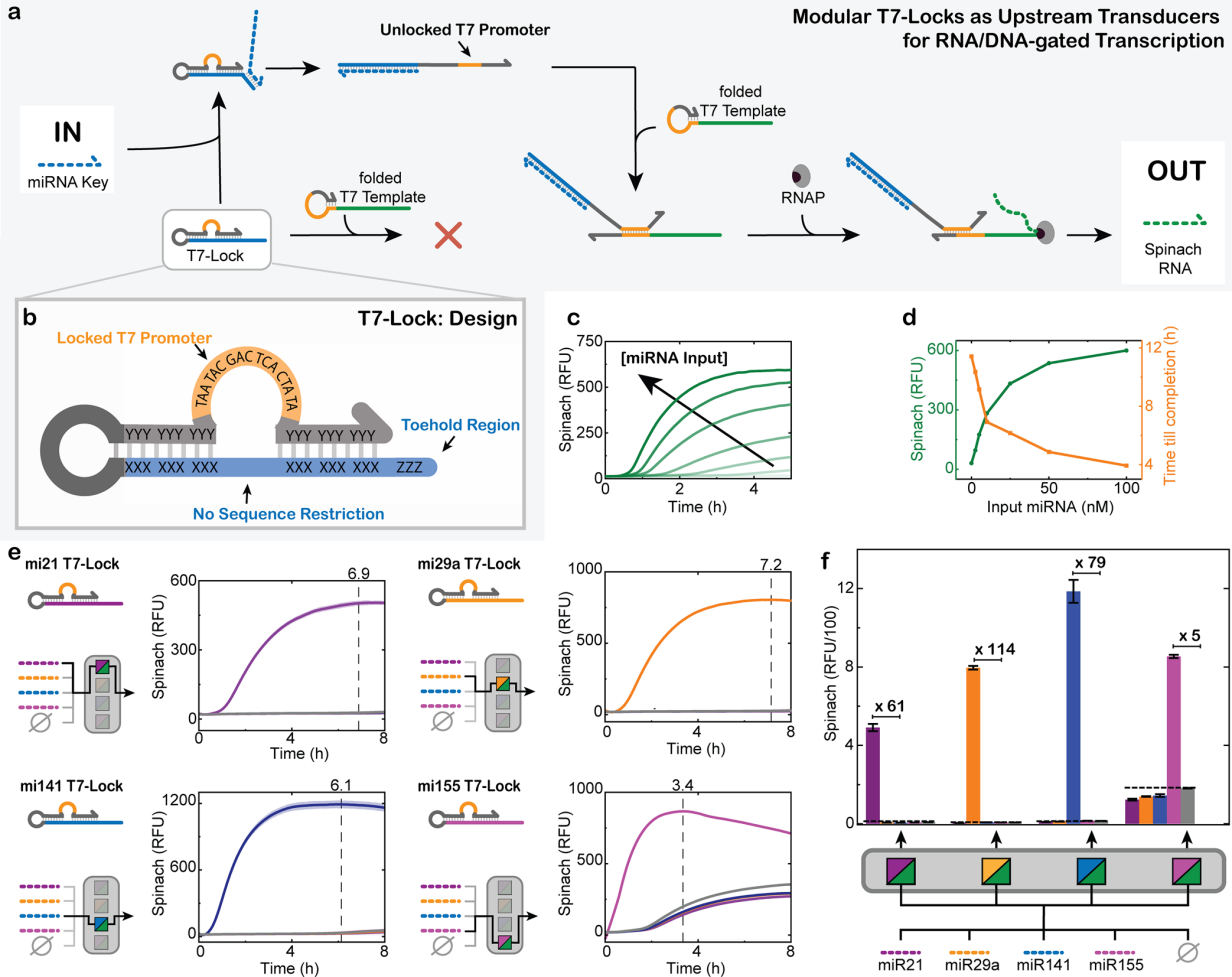
T7-Lock enables miRNA-triggered transcription. (a) System design where the T7 promoter is thermodynamically sequestered in the T7-Lock, preventing it from hybridizing to the transcription template. Upon addition of a miRNA key strand, the T7-Lock opens by an SDR, exposing the T7 promoter. The T7 promoter consecutively binds to the template, initiating transcription. The transcribed Spinach RNA binds to DFHBI, inducing a fluorescent signal. (b) Modular T7-Lock design. The displacement domain has no sequence restriction, allowing customization with freely designable oligonucleotide keys. (c) Real-time fluorescence measurement of miRNA-141-triggered transcription at varying concentrations of miRNA-141. Conditions: HiScribe T7 Quick High Yield RNA Synthesis Kit, 1.25 μM folded Spinach template, 25 nM mi141-T7 Lock, 40 mM HEPES, 125 mM KCl, 5 mM MgCl_2_, 62.5 mM NaCl, and 5 μM DFHBI at 37 °C. Input: 0, 2.5, 5, 10, 25, 50, or 100 nM miRNA 141. Single measurements shown. (d) Dependence of transcription yield (Spinach, RFU) and reaction-completion time on miRNA-141 concentration with data obtained from the curves in panel (c). (e) Selective detection of miRNA input, using a mi21 T7-Lock, mi29a T7-Lock, mi141 T7-Lock, or a mi155 T7-Lock. To each T7-Lock, miRNA-21, 29a, 141, and 155 were added separately, after which transcription of the Spinach aptamer was recorded. Fluorescent signals above blank level are only observed for the correct miRNA input. Conditions: HiScribe T7 Quick High Yield RNA Synthesis Kit, 1.25 μM folded Spinach template, 25 nM miX-T7 Lock, 40 mM HEPES, 125 mM KCl, 5 mM MgCl_2_, 62.5 mM NaCl, and 5 μM DFHBI at 37 °C. Input: 250 nM miRNA X, or 0 nM miRNA X (blank) (*n* = 5; shaded areas (partly invisible as too small) represent the standard deviation). (f) Overview of the output RFU maxima from each T7-Lock, along with correct and false miRNA keys (from panel (e)). The bar chart shows the average of 5 measurements along with the corresponding standard deviation.

Taking miRNA-141 as a first example, real-time fluorescence measurements confirm that miRNA-141 effectively triggers transcription, with reaction time and product yield scaling proportionally to the input miRNA-141 concentration (Figure 3c,d). The correlation between input and output enables quantitative miRNA detection. In the absence of the key strand, no transcription takes place, confirming once again high ON/OFF ratios.

To validate the universality of our design, we constructed four distinct T7-Locks, each responsive to a different miRNA sequence (miRNA-21, miRNA-29a, miRNA-141, and miRNA-155; sequences listed in Table S1). The X/Y sequences are designed to cover the entire miRNA sequences with 3 nts on the Y stretch serving as toehold. Figure 3e shows that transcription is specifically activated only by the corresponding miRNA keys (color-coded for clarity). Occasional leakage is observed, as for the miRNA-155 T7-Lock. However, even in this case, the transcriptional readout is never greater for a false miRNA compared to the blank (nothing added). This means that this leakage stems from poor sequence compatibility between T7-Lock and the template, rather than from a side reaction between T7-Locks and nonspecific miRNAs. In fact, NUPACK simulations show that a small quantity of each T7-Lock is predicted to interact with the Spinach template while no miRNA is present.^55^ Though almost negligible for the mi21-, mi29a-, and mi141-T7 Lock, some higher degree of unsolicited duplex formation between the mi155 T7-Lock and the template explains the occurrence of some transcription in the blank sample and the lower ON/OFF ratio (see Figure S5 for NUPACK calculations). Even though this residual transcriptional activity is perfectly fine for selective miRNA detection, we chose to include this example to show that some further sequence optimization for some inputs would be needed to reach full dormancy if desired for other applications.

Overall, the results demonstrate high specificity and minimal cross-interference among the designs. Figure 3f quantifies the transcription output for each T7-Lock at the time point of maximum signal following the addition of its matching miRNA key. Negative controls (false miRNA) for all designs never exceed the blank (where no miRNA has been added), confirming orthogonality across sets. Interestingly, the transcription efficiency varies with the T7-Lock–miRNA pairs. We attribute this to differences in the ability of miRNA inputs to form different secondary structures, that may influence the rate and efficiency in T7-Lock opening.^56^ The miRNA-21, miRNA-29a, and miRNA-141 T7-Locks have remarkable ON/OFF ratios of 61, 114, and 79, respectively. miRNA-155 has a slightly lower ON/OFF ratio of 5.

We further tested each lock with DNA analogs (miDNA) matching the miRNA sequences. In all cases, transcription is triggered even more efficiently by miDNA strands, with ON/OFF ratios of 113, 432, 410, and 21 for miDNA-21, −29a, −141, and −155, respectively (Figure S6). In summary, the T7-Lock enables sequence-specific transcription initiation by a broad range of oligonucleotide triggers, including both miRNA and miDNA, while maintaining high specificity and orthogonality. These features make the system a robust platform for nucleic acid sensing and DNA-based computation.

### Signal Amplification via Positive Feedback Integration

Although T7 RNAP generates multiple RNA copies from a single template, and should in principle be limited by the available NTPs with respect to the total product generation, a persistent correlation between [input RNA] and [output RNA] can be observed (Figure 3c,d). Even though this [input]-controlled behavior is advantageous for quantitative analytical applications, we asked the question whether it is possible to enhance the signal strength by the incorporation of a positive feedback loop.

A positive feedback loop requires the design of a system where the output signal from transcription is identical to the input. A key challenge in implementing this concept lies in the fact that the transcription template must be fully complementary to both the input and output strands. This leads to a scenario in which the input miRNA preferentially hybridizes with a ssDNA template rather than opening the T7-Lock, resulting in transcriptional dormancy—a side reaction illustrated in Figure S7. In principle, this type of dormancy may be prevented by using a dsDNA template. However, when aiming at making short 19–24 nt long transcripts for light-up aptamers and considering the operation temperature of 37 °C, the corresponding strands of the dsDNA template would be in a dynamic hybridization equilibrium. Thus, even in excess of T7-Locks, the miRNA inputs would find an opened dsDNA template allowing the stronger RNA-DNA hybridization to dominate, leading again to a scavenging of the miRNA input at the wrong position.^57^ At minimum sequence length, a hairpin structure that folds back onto the T7 template may help to prevent this unwanted RNA blocking of the transcription region of the template. This would however lead to transcription around the hairpin, ultimately furnishing a stable hairpin RNA that cannot open additional T7-Locks to reach autocatalytic amplification.

To tackle these issues, we developed a facile strategy to terminate transcription in the middle of a template, enabling precise control over transcript length without compromising design flexibility. To our knowledge, previous studies only reported the site-controlled termination of transcription by incorporating specific sequences and/or secondary structures in the templates.^58,59^ In those cases, the freedom of the template sequence has to be compromised to conform to these termination motifs. In contrast, we simply introduce a C12 spacer (−(CH_2_)_12_−) to abruptly terminate transcription (Figure 4a). The C12 spacer is a commercially available DNA modification that can be inserted between nucleotides.

**Figure 4:**
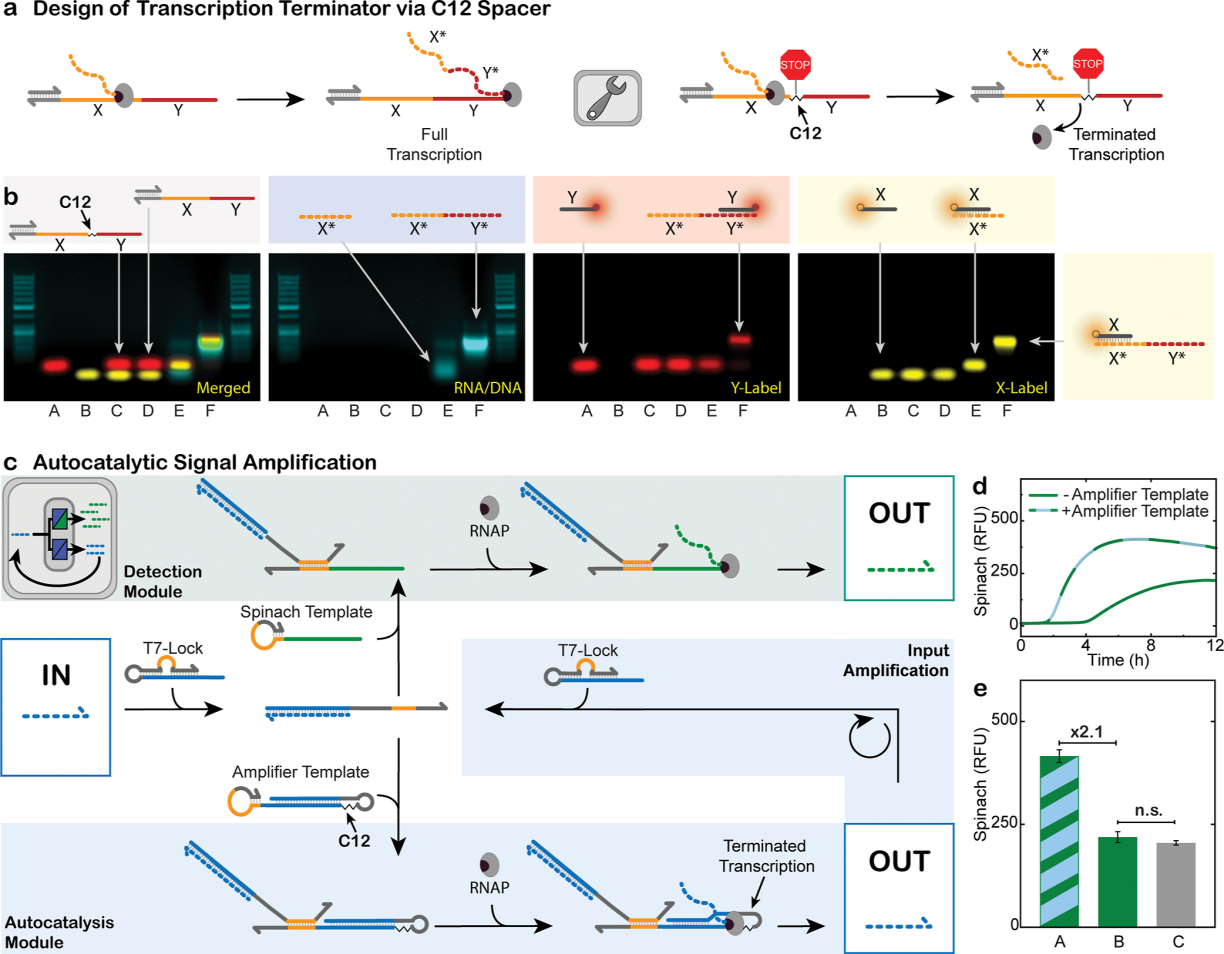
C12 spacers are efficient transcription terminators and enable autocatalytic signal amplification. (a) Schematic representation of site-specific transcription termination caused by an internal C12 spacer modification in the template. (b) AGE: Fluorescence images of RNA products, templates and probe strands to characterize transcribed products with or without a C12-modified template. (A): DY-647 labeled Y-Marker, (B): Atto-565 labeled X-marker, (C): X-Marker, Y-Marker, and C12-modified template, (D): X-Marker, Y-Marker, and unmodified template, (E): X-Marker, Y-Marker, and RNA transcribed from C12-modified template, (F): X-Marker, Y-Marker, and RNA transcribed from unmodified template. 50 bp DNA ladders as reference. (c) Schematic representation of a miRNA amplification circuit. Next to the standard folded Spinach template, an amplifier template is added in a substoichiometric amount. The amplifier template has a C12 spacer to only transcribe a sequence that is equal to the input. The transcribed sequence in turn opens more T7-Locks, causing an increased transcription of Spinach. (d) Real-time fluorescence measurements of a miRNA 141-triggered transcription sample containing only a Spinach template, or a Spinach template plus the C12-modified amplifier template (100:1 ratio, respectively). After addition of 100 nM miRNA-141, the sample with the C12-modified amplifier transcribes 2.1× more product at a faster rate. Conditions: HiScribe T7 Quick High Yield RNA Synthesis Kit (NEB), 1.25 μM folded Spinach template, 0 nM (-Amplifier) or 12.5 nM (+Amplifier) C12 modified amplifier template, 12.5 nM mi141-T7 Lock, 40 mM HEPES, 125 mM KCl, 5 mM MgCl_2_, 62.5 mM NaCl, and 5 μM DFHBI at 37 °C. Input: 100 nM miRNA 141 (*n* = 5; shaded areas (partly invisible as too small) represent the standard deviation). (e) Bar chart summarizing maximum intensity data of (d): (A) Spinach template + autocatalytic amplifier template; (B) Spinach template; (C) Further control: Spinach template + “amplifier template” without C12 modification. The addition of an unmodified amplifier strand has no significant effect on the transcription yield. Averages and error bars as standard deviations are derived from panel (d). n.s. = not significant as determined by a two-tailed Student’s *t*-test *P* = 0.06 (n.s.: *P* > 0.05).

As proof of concept, we designed two simple linear ssDNA transcription templates. Our C12-modified template contains an X and a Y sequence separated by a C12 spacer, while the control lacks the spacer, positioning X directly adjacent to Y. Following transcription, the products were allowed to hybridize with dye-labeled Atto 565-X (yellow channel) and DY-647-Y ssDNA (red channel) complementary to the X* or Y* sequence of the transcribed RNA. Native agarose gel electrophoresis (AGE) analysis with ROTI staining reveals that the unmodified control template produces a significantly longer transcript than the C12-modified variant (see migration distances in lanes F vs lane E in AGE; Figure 4b). Both RNA transcripts hybridize with Atto-565-X, confirming the presence of X* in the transcripts. Notably, only the unmodified control produces transcripts that also hybridize with DY-647-Y, whereas the C12-modified template does not. This confirms that the C12 spacer serves as an effective site-specific transcription terminator. In contrast, integration of a shorter C3 spacer in the template results in less effective termination, as a substantial amount of RNA containing the full X*–Y* sequence is detected after transcription (Figure S8). We hypothesize that the ∼20 nt-long transcription footprint of the T7 RNA polymerase can partially tolerate a short C3 defect, whereas the longer C12 spacer introduces a gap too large to be bridged, thereby preventing transcriptional readthrough.^32,33^

The C12 transcription terminator can be leveraged for an amplification circuit because now it is possible to fold an extended sequence as a hairpin back onto the template. This efficiently blocks unwanted binding of the input miRNA (the key) to the template, yielding an amplification module that provides an output identical to the input. The additionally generated outputs serve as keys to unlock more T7-Locks. Figure 4c shows schematically the combination of the autocatalytic amplifier module (bottom) with the reporter module (Spinach, top) for enhanced signal generation. We chose to operate at low amplifier and T7 Lock concentrations to highlight the impact on amplification, as well as have an excess of template to ensure efficient binding of the opened T7 Lock to the templates. Figure 4d visualizes the effect of this amplification for sensing miRNA-141. For the detection of 100 nM miRNA, the presence of the amplification module at a 1:100 ratio relative to the detection module leads to a much stronger fluorescence increase, along with an almost halved response time to reach maximum fluorescence intensity of 6.9 h in contrast to the original 13.2 h. Figure 4e quantifies the fluorescence intensity maxima of Spinach transcribed in the presence or absence of a C12-modified amplifier (A vs B) to a 2.1-fold signal increase when using the autocatalytic transducer. A further control—a nonmodified hairpin amplifier template devoid of the C12 modification (C)—does not lead to this amplification because all the produced RNA folds into a hairpin and is unable to function as input (gray bar in Figure 4e). Taken together, this highlights the critical role of the C12 spacer in enabling deliberate site-specific termination and effective autocatalytic signal amplification.

### miRNA-Triggered Protein Expression Gated on a Transcriptional Level

Having demonstrated that our toolbox accommodates diverse oligonucleotide inputs, we next show its capacity to generate a protein output. While small miRNAs in cells primarily function in post-transcriptional silencing, synthetic networks would equally benefit from promoted gene expression.^31^ To this end, prior studies exploiting small RNAs to trigger protein expression have predominantly relied on engineering riboswitches in prokaryotic systems, or modifying internal ribosome entry sites (IRES) in eukaryotic mRNA to enable miRNA-promoted translation.^60–67^ These approaches operate on a translational level and require tailored mRNA designs depending on the downstream mRNA/protein sequence and optimization for a specific trigger miRNA. Additionally, by operating on a mRNA level, one miRNA strand only activates one mRNA strand, thus requiring to consider stoichiometric trigger ratios. In contrast, we can leverage our T7-Lock/key technology for gating mRNA transcription from DNA, hence installing the miRNA processing on a transcriptional level upstream of mRNA translation. This enables the synthesis of multiple active mRNA copies from one single miRNA trigger. Control on the DNA level also overcomes the need to engineer the mRNA (e.g., at the IRES) to make it compatible with a specific miRNA trigger.^60–63^

For miRNA-triggered transcription of mRNA from a long gene, a key requirement is a DNA template that includes an incomplete T7 promoter binding site lacking its complementary T7 promoter. This necessitates a free ssDNA overhang at the 3′ end of the gene, for which we developed a facile, dedicated PCR protocol (Figure 5a). We selected the *mCherry* gene as its translation can be easily monitored by fluorescence. To generate the necessary 3′ end overhang, we extracted and amplified the gene from a plasmid by PCR. Building on our transcription terminator in Figure 4, we hypothesized that, with the short template–enzyme interaction region of *Taq* polymerase, a short C3 or C12 spacer would work equally well for site-specific termination of a DNA polymerase chain (PCR) reaction, prompting us to use C3 or C12-modified primers.^68^ Subsequent copy/amplification cycles yield a dsDNA gene with a 5′ ssDNA overhang. Using a T4 DNA ligase, this overhang can be ligated with a partially complementary ssDNA containing a T7 promoter binding site. This results in a construct with a 3′ overhang, where the DNA strand that is read out by the T7 RNAP (bottom strand) does not contain any Cx spacer. Thus, T7 RNAP-mediated transcription should not be hampered once the promoter comes in, but at the same time become conditional on the promoter being present.

**Figure 5:**
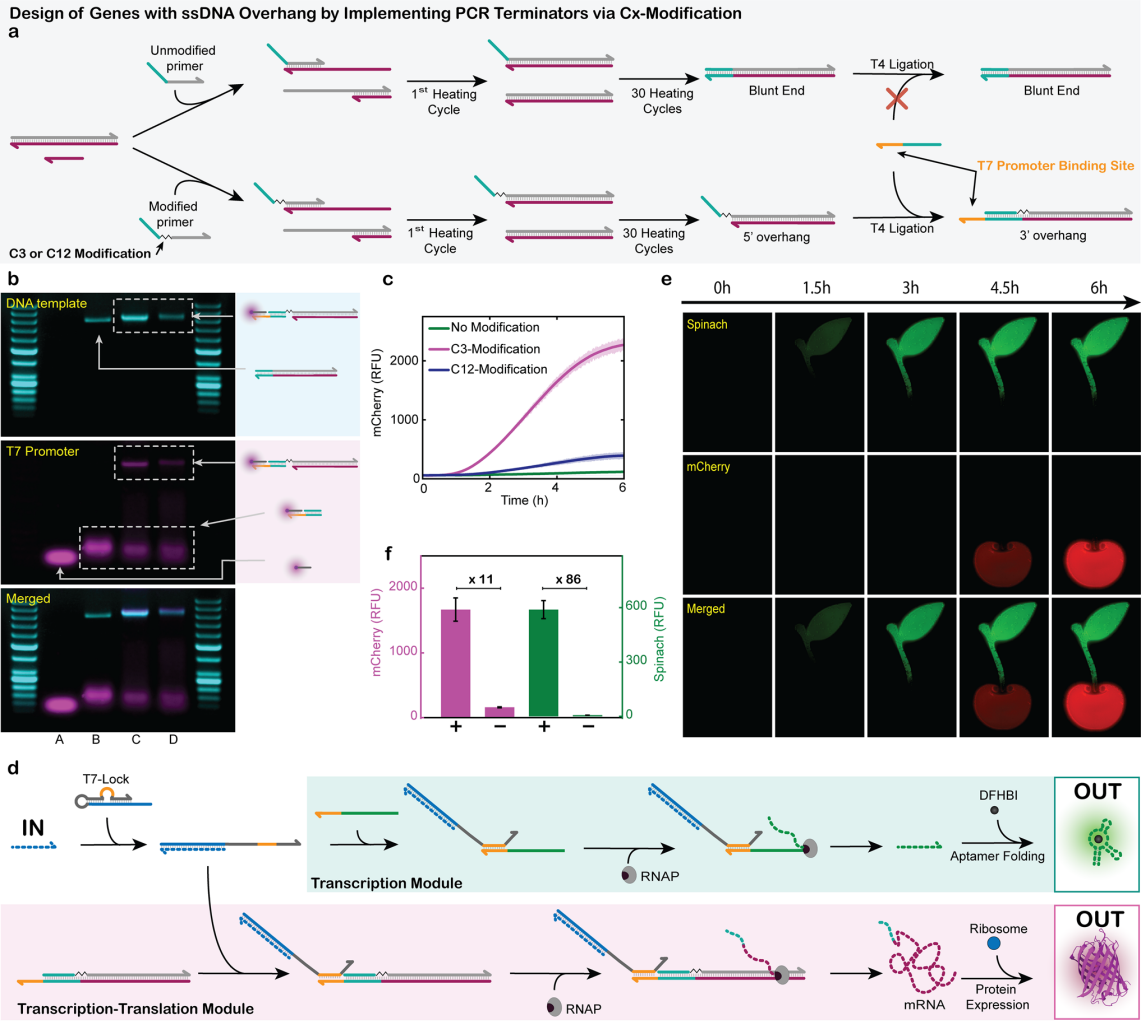
miRNA-triggered protein expression using T7-Locks. (a) Schematic of the PCR strategy to generate DNA templates with a 3′ overhang. Top: Full incorporation of a sticky-end primer produces a blunt-end product. Bottom: A C3 spacer between primer and sticky-end blocks extension, yielding a 5′ overhang that mediates ligation of a ssDNA strand containing a T7 promoter binding site. (b) AGE: Fluorescence images of PCR products: (A): blank containing fluorescently labeled promoter; (B): product after PCR with unmodified primer; (C): PCR product with C3-modified primer; (D): PCR product with C12-modified primer. Each PCR product was mixed with a DY-647 P1 labeled T7 promoter, and complementary ssDNA with a T7 promoter binding site. Interactions with the fluorescent promoter are observed only for the PCR products containing the C3- and C12-modified primer. 50 bp DNA ladders as internal references. (c) Direct T7 promoter-triggered transcription and translation of *mCherry* using templates from (b). C3-modified templates yield the strongest fluorescence signal; C12 yields less; unmodified templates show no signal. Conditions: 200 nM purified PCR product (*mCherry* template) with 1.25 μM T7 promoter, in a PURExpress Kit (NEB) at 37 °C (*n* = 3; shaded areas (partly invisible as too small) represent the standard deviation). (d) Schematic showing how a single miRNA input and T7-Lock can initiate either direct RNA transcription of fluorescent Spinach, or mRNA transcription followed by protein translation of *mCherry*. (e) Fluorescence images of miRNA 141-triggered transcription of the Spinach aptamer and translation of the *mCherry* protein over 6 h. Conditions Spinach transcription: HiScribe T7 Quick High Yield RNA Synthesis Kit, 1.25 μM folded Spinach template, 12.5 nM mi141-T7 Lock, 40 mM HEPES, 125 mM KCl, 5 mM MgCl_2_, 62.5 mM NaCl, and 5 μM DFHBI at 37 °C. Input: 250 nM miRNA 141. *mCherry* translation: 200 nM purified PCR product, 12.5 nM mi141 T7-Lock, and 250 nM miRNA 141 in a PURExpress Kit (NEB) at 37 °C. (f) Maximum *mCherry* and Spinach RFU of curves from plate-reader fluorescence measurements in the presence or absence of miR141. The mCherry translation has an ON/OFF ratio of 11, and Spinach transcription of 86. Conditions: identical to panel (e). Averages and error bars are derived from *n* = 4 measurements.

Following this rationale, we generated three variants of the PCR product: (1) C3-modified 3′ overhang gene, (2) C12-modified 3′ overhang gene, and (3) a classically amplified gene as control using nonmodified primers and leading to blunt ends. AGE analysis first shows that similar-length genes are generated in all three PCR reactions (Figure 5b, DNA stain, cyan channel). This suggests that the C3 and C12 spacers do not interfere with the basic PCR amplification process. More importantly, the hybridization of the overhang with a dye-labeled T7 promoter/binding site construct (magenta channel) clearly confirms the successful integration of the 5′ ssDNA overhangs in the C3 and C12-modified constructs via the appearance of colocalized bands in the magenta channel. The unmodified control does not bind the dye-labeled T7 promoter due to its blunt end nature. This confirms the successful generation of sticky-end PCR products with 3′ overhangs using C3 and C12 spacers in the primers.

We next tested how these three PCR products—after ligation with a T7 promoter binding site—perform in protein synthesis after the addition of free T7 promoter using a reconstituted cell-free TX-TL kit. Figure 5c shows that the C3-modified gene (1) produces the brightest fluorescence, indicating efficiently triggered transcription and translation. No expression can be found for the unmodified gene (3) that lacks the T7 promoter binding site sequence and thus cannot be triggered for transcription. The C12-modified gene (2) is active for translation but yields a significantly weaker signal compared to the C3-analog (1). We associate this with inefficient ligation of the T7 promoter binding site across the C12 junction to the 5′ overhang of the PCR product rather than with inefficient transcription of the final construct.

Finally, according to the schematics in Figure 5d, we assembled the C3-modified *mCherry* gene with its ligated promoter recognition site, together with a T7-Lock that can be opened selectively by the miRNA-141 key, and investigated the gated TX-TL signal transduction. In parallel, to directly compare the reaction kinetics with the light-up aptamer, we also assembled a miRNA-141-gated transcription system. To visualize the macroscopic differences in kinetics between the two pathways, we loaded the samples into a cherry-shaped mold, with the Spinach aptamer transcription mixture in the leaf and the *mCherry* TX-TL in the fruit (Figure 5e). Upon addition of 250 nM miRNA-141, fluorescence imaging shows a sequential light-up of both compartments (Movie S1). The Spinach aptamer is transcribed in less than 1.5 h, and the reaction reaches completion in 3 h. In contrast, the translation of *mCherry* has a delayed onset and requires a minimum of 6 h to complete. The delay signifies the time required for the mRNA to be translated and folded into a functional protein. A parallel plate reader experiment quantifies the maximum obtained fluorescence in presence and absence of miRNA-141 (Figure 5f). The ON/OFF ratios of *mCherry* and Spinach of 11 and 86, respectively, signify high dormancy and efficient specific activation. Critically, these results validate the functionality of the T7-Lock tool even in a complex cell-free TX-TL environment that comprises a range of proteins, ribosomes, as well as NTPs and tRNAs to enable protein synthesis. This highlights the modularity of the toolbox in pushing miRNA-triggered transduction cascades up to the level of protein expression.

## Conclusion

We have developed a modular and low-leakage T7 RNA polymerase (T7 RNAP) toolbox that enables programmable signal transduction from oligonucleotide sensing to protein expression. At the core of this system is the T7-Lock—a thermodynamically sequestered promoter architecture—which has been designed to activate transcription exclusively upon recognition of sequence-specific miRNA or DNA inputs. This modular architecture allows broad input compatibility without compromising specificity or orthogonality.

We demonstrated high-fidelity miRNA detection using Spinach aptamer readouts, achieving ON/OFF ratios exceeding 100 and enabling parallel detection of multiple miRNA species with minimal crosstalk. To amplify weak input signals, we further implemented autocatalytic feedback loops using site-specific transcription termination via a C12 spacer, enabling robust amplification while suppressing background leakage. This C12 spacer addition to a transcription template is a termination feature of generic value for other applications surrounding polymerases. Here it allowed the synthesis of an RNA equal to the input at a minimum energy expense of NTPs because the transcribed template can be kept as short as needed.

To bridge transcription and translation, we further established a PCR protocol to generate sticky-end gene templates with ssDNA overhangs—now leveraging the Cx spacers to terminate the PCR reaction—allowing for miRNA-triggered mRNA transcription and downstream protein expression in cell-free TX-TL systems. Since such ssDNA overhang-bearing genes are broadly relevant for gene immobilization, such as in hydrogels, on surfaces, or within synthetic cells, this facile method might find broader applications, especially since current strategies for generating long genes with ssDNA anchors, such as those available from asymmetric PCR, lambda or T7 exonuclease digestion, and biotin–streptavidin pulldown, remain rather low-yield and costly.^69–73^ The successful demonstration of miRNA-gated protein expression underscores the robustness of our systems within complex media.

Looking forward, the versatile T7 RNAP modalities generated in this study offer a powerful addition to the existing T7 RNAP circuitry, enabling facile construction of dynamic CRNs with embedded logic, amplification, and multilevel responses.^20,22,24–30^ Its minimal sequence constraints, minimum energy footprint, and biochemical compatibility make it well-suited for integration into cellular systems, where programmable RNA sensing and actuation could potentially be coupled to native gene expression. For mammalian cells, this would require bringing the T7 RNAP along in a nanocarrier or supplying a cell with a plasmid coding for T7 RNAP production in situ. The principle operation of T7 RNAP inside mammalian cells has however been demonstrated before.^74^ For successful integration, stabilization of the DNA components, e.g., through backbone modifications, should be considered to prevent nuclease-mediated degradation. In addition, optimizing inter- and intramolecular hybridization may be necessary to accommodate the conditions (e.g., crowding, ionic strength) within the cellular environment. Taken together, this platform lays the groundwork for future applications in in vivo biosensing, therapeutic circuits, and synthetic biology in general.

## Supplementary Material





## Notes

Materials and instrumentation, experimental details, data analysis methods, and supplementary figures (PDF)
Movie S1: miRNA activated transcription and translation (MP4)
