# Supplementary material for: A Modular T7 RNA Polymerase Toolbox Linking Selective miRNA Detection to Signal Amplification and Protein Expression
Source: J Am Chem Soc. 2025 Nov 19;147(48):44468–78. doi: 10.1021/jacs.5c16214 (PMC12679629; doi:10.1021/jacs.5c16214)
Supplement: Supplementary file 1 [file ja5c16214_si_001.pdf]

Supporting Information

A Modular T7 RNA Polymerase Toolbox Linking Selective miRNA Detection to Signal Amplification and Protein Expression

Maria Vonk-de Roy[1] and Andreas Walther[1*]

[1]Life-Like Materials and Systems, University of Mainz, Duesbergweg 10-14, 55128 Mainz, Germany.
[*]Corresponding author: andreas.walther@uni-mainz.de

Contents

1. Materials......2
2. General Characterization Methods & Instruments......2
3. Methods......3
   Transcription of Spinach Aptamer......3
   miRNA-Triggered Transcription......4
   miRNA Signal Amplification Circuit......5
   miRNA Triggered Protein Expression......5
4. Supplementary Table......8
   Table S1: Oligonucleotide sequences......8
5. Supplementary Figures......10
   Figure S1. Secondary structure linear template vs folded template.......10
   Figure S2. Interactions of T7 RNAP with folded template vs linear template + T7 promoter......11
   Figure S3. Dependency of T7 promoter concentration on quantity RNA transcribed.......12
   Figure S4. Transcriptional activity following prolonged dormancy periods.......13
   Figure S5. Hybridization between T7-Locks and Spinach template in absence of miRNA......14
   Figure S6. Selective detection of miDNA input......15
   Figure S7. Limitations of internal signal amplification template.......16
   Figure S8. Limited transcription termination with C3 modification in template.......17
6. References......18

1. Materials

HEPES (≥99.5%), n-hexadecane (99.0%), potassium chloride (99%), and magnesium chloride solution (BioUltra, 2 M in H_2O) were purchased from Sigma Aldrich. ROTIPHORESE®50x TAE buffer and ROTI®GelStain (20,000x conc) were purchased fromfrom Carl Roth. GeneRuler 50 bp DNA ladder, 1x TE Buffer (10 mM Tris-HCl (pH 8.0), 0.1 mM EDTA), and UltraPure™ Glycerol from Thermo Fisher Scientific. 5Z-[(3,5-difluoro-4-hydroxyphenyl)methylene]-3,5-dihydro-2,3-dimethyl-4H-imidazol-4-one (98%,DFHBI) from Hycultec. Agarose, hydrochloric acid (0.1 N, Titripur) and sodium chloride (≥98%, technical) from VWR. All chemicals were used as received without further purification.

Taq polymerase (5,000 units/mL),10x Standard *Taq* Buffer (100 mM Tris-HCl, 500 mM KCl, 15 mM $MgCl_2$, pH 8.3 @25 °C), deoxynucleotides (dNTP) solution mix (10 mM each), T4 DNA ligase (400,000 units/mL), 10x T4 DNA ligase buffer (500 mM Tris-HCl, 100 mM MgCl2, 10 mM ATP, 100 mM DTT, pH 7.5 @25 °C), HiScribe® T7 Quick High Yield RNA Synthesis Kit (T7 Polymerase Mix and NTP Buffer Mix, E2050L), PURExpress® *In Vitro* Protein Synthesis Kit (E6800L), RNase Inhibitor Murine (40,000 units/mL (M0314S), and Nuclease-free Water were purchased from New England Biolabs, and used according to the manufacturer's protocol (unless stated differently).

Plasmid OSCA1.1 (catalog #136592) was purchased from Addgene. All other oligonucleotides (with sequences listed in Table S1) were purchased from Biomers GmbH and dissolved in Nuclease-free water. All buffers, DNA and RNA stock solutions, and synthesized DNA were dissolved in Nuclease-free water as well. 10x Spinach Buffer (400 mM HEPES, 1.25 M KCl and 50 mM $MgCl_2$, pH 7.4 @ 25 °C) was prepared in-house.

2. General Characterization Methods & Instruments

Real-time Fluorescence Measurements

Real-time fluorescence measurements were recorded on a TECAN (SPARK control v3.1) microplate reader with excitation and emission wavelengths for the Spinach aptamer at 447 and 501 nm and a bandwidth of 20 nm. *mCherry* was detected with the excitation set at 585 and the emission at 610 nm, with bandwidths of 10 nm. All samples were prepared in a volume of 20 µL (unless stated differently), put in a Corning® 384-well clear-bottom polystyrene plate with a non-binding surface, and covered with 10 µL hexadecane. All measurements were performed at 37 °C.

NUPACK Simulations

All simulations were run in NUPACK with the following conditions: 125 mM NaCl, 6 mM $MgCl_2$ and 37 °C.

Data Processing

The plotted curves represent an average of a number of measurements listed in the figure captions and methods section below. The shaded regions around the curve represent standard deviations calculated in OriginPro 2023 using the 'SD Err' function. All (mean) curves were smoothed in OriginPro 2023 using the Savitzky-Golay method (50 points of window, polynomial order 2) to reduce noise.

3. Methods

Transcription of Spinach Aptamer

Directly triggered transcription was performed by assembling a master mix with a volume of 270 µL. To a 500 µL PCR tube, 100 µL NTP Buffer Mix, 20 µL T7 RNA Polymerase Mix, 30 µL DFHBI (50 µM, in DMSO), 30 µL 10x Spinach Buffer, 3.75 µL linear Spinach Template (100 µM), 18.75 µL NaCl (1000 mM), and 67.5 µL Nuclease-free water were added, followed by a thorough homogenization with a pipette. Of the master mix, 18 µL was aliquoted into 15 wells of a 384-well plate. To those wells 2 µL T7 promoter DNA (12.5 µM), T7 promoter RNA (12.5 µM), or miDNA 141 (12.5 µM) were added in quintuplicates. The samples were overlaid with hexadecane and measured in the plate reader. *The final reaction volume of each sample was 20 µL and contained 6.67 µL NTP Buffer Mix, 1.33 µL T7 RNA Polymerase Mix, 5 µM DFHBI, 40 mM HEPES, 125 mM KCl, 5 mM $MgCl_2$, 1.25 µM linear Spinach Template, 62.5 mM NaCl, and 1.25 µM of the trigger (T7 promoter DNA, T7 Promoter RNA, or miDNA141).*

Parasitism of the folded vs linear spinach template was assessed by assembling a master mix with a volume of 180 µL. To a 200 µL PCR tube, 66.7 µL NTP Buffer Mix, 13.3 µL T7 RNA Polymerase Mix, 20 µL DFHBI (50 µM, in DMSO), 20 µL 10x Spinach Buffer, and 47.5 µL Nuclease-free water were added. The master mix was homogenized and aliquoted (10x18 µL) into a 384-well plate. To those aliquots, 2 µL of a linear Spinach Template stock (3.1, 6.3, 12.5, 25 or 50 µM), or 2 µL of a folded Spinach Template stock (3.1, 6.3, 12.5, 25 or 50 µM) were added. The samples were overlaid with hexadecane and measured in the plate reader. *The final reaction volume of each sample was 20 µL and contained 6.67 µL NTP Buffer Mix, 1.33 µL T7 RNA Polymerase Mix, 5 µM DFHBI, 40 mM HEPES, 125 mM KCl, 5 mM $MgCl_2$, 62.5 mM NaCl, and the linear or folded Spinach Template in varying concentrations (0.31, 0.62, 1.25, 2.5 or 5.0 µM).*

The triggered activity of the folded vs linear spinach template was performed by assembling a master mix with a volume of 72 µL. To a 200 µL PCR tube, 26.7 µL NTP Buffer Mix, 5.3 µL T7 RNA Polymerase Mix, 8 µL DFHBI (50 µM, in DMSO), 8 µL 10x Spinach Buffer, 5 µL NaCl (1000 mM), 1 µL T7 promoter DNA (100 µM), and 18 µL Nuclease-free water were added. The master mix was homogenized and aliquoted (4x18 µL) into a 384-well plate. To those aliquots, 2 µL of a linear Spinach Template stock (12.5 µM), or 2 µL of a folded Spinach Template stock (12.5 µM) were added. The samples were overlaid with hexadecane and measured in the plate reader. *The final reaction volume of each sample was 20 µL and contained 6.67 µL NTP Buffer Mix, 1.33 µL T7 RNA Polymerase Mix, 5 µM DFHBI, 40 mM HEPES, 125 mM KCl, 5 mM $MgCl_2$, 62.5 mM NaCl, 1.25 µM T7 promoter DNA, and 1.25 µM linear or folded Spinach Template.*

The relationship between RFU of the transcribed Spinach and concentration of T7 promoter (DNA) was established by first assembling a master mix with a volume of 144 µL total. To a 200 µL PCR tube, 53.4 µL NTP Buffer Mix, 10.6 µL T7 RNA Polymerase Mix, 16 µL DFHBI (50 µM, in DMSO), 16 µL 10x Spinach Buffer, 2 µL folded Spinach Template (100 µM), 10 µL NaCl (1000 mM) and 36 µL Nuclease-free water were added. The master mix was homogenized and aliquoted (8x18 µL) into a 384-well plate. To those aliquots either 2 µL Nuclease-free water (negative control), or 2 µL of a T7 promoter stock (10, 50, 100, 250, 500, 1000, or 1500 nM) was added. The samples were overlaid with hexadecane and measured in the plate reader. *The final reaction volume of each sample was 20 µL and contained 6.67 µL NTP Buffer Mix, 1.33 µL T7 RNA Polymerase Mix, 5 µM DFHBI, 40 mM HEPES, 125 mM KCl, 5 mM $MgCl_2$, 1.25 µM folded Spinach Template, 62.5 mM NaCl, and 0, 1, 5, 10, 25, 50, 100 or 250 mM T7 promoter DNA.*

The activity after dormancy of the T7-promoter (DNA) triggered transcription was determined by first assembling a master mix with a volume of 108 µL total: To a 200 µL PCR tube, 40 µL NTP Buffer Mix, 8 µL T7 RNA Polymerase Mix, 12 µL DFHBI (50 µM, in DMSO), 12 µL 10x Spinach Buffer, 1.5 µL folded Spinach Template (100 µM), and 34.5 µL Nuclease-free water were added. The master mix was homogenized and aliquoted (6x18 µL) into a 384-well plate. The samples were overlaid with hexadecane and measured in the plate reader. During analysis, 2 µL of a 12.5 µM T7 promoter stock was added to each well at t = 0, 2, 3, 4, 5 or 6 hours. *After completion, the final reaction volume of each sample was 20 µL and contained 6.67 µL NTP Buffer Mix, 1.33 µL T7 RNA Polymerase Mix, 5 µM DFHBI, 40 mM HEPES, 125 mM KCl, 5 mM $MgCl_2$, 1.25 µM folded Spinach Template, and 1.25 µM T7 promoter DNA.*

miRNA-Triggered Transcription

Snap cooling of T7-Lock strands was done in 200 µL PCR tubes and reaction volumes of 100 µL. The T7-Lock strands were dissolved at 100 nM or 50 nM in 250 mM NaCl in Nuclease-free water. The samples were snap cooled in a qTower[3] Real-time Quantitative PCR (Analytik Jena) by heating the samples from 20 °C to 95 °C (8.0 °C/s), holding them at 95 °C for 60 seconds, followed by a rapid cooling to 4 °C (8.0 °C/s), where the samples were held for at least 5 minutes. For each transcription experiment a freshly snap-cooled T7-lock sample was prepared.

Quantitative miRNA detection was established by first assembling a master mix with a volume of 126 µL in total: To a 200 µL PCR tube, 46.7 µL NTP Buffer Mix, 9.3 µL T7 RNA Polymerase Mix, 14 µL DFHBI (50 µM, in DMSO), 14 µL 10x Spinach Buffer, 1.75 µL folded Spinach Template (100 µM), and 5.25 µL Nuclease-free water were added. The master mix was briefly homogenized with a pipette. Afterward, 35 µL of a freshly snap-cooled mi141 T7-Lock (100 nM, in 250 mM NaCl) was added. The master mix was homogenized and aliquoted (7x18 µL) into a 384-well plate. To those aliquots either 2 µL Nuclease-free water (negative control), or 2 µL of a miRNA 141 stock (25, 50, 100, 250, 500, or 1000 nM) was added. The samples were overlaid with hexadecane and measured in the plate reader. *The final reaction volume of each sample was 20 µL and contained 6.67 µL NTP Buffer Mix, 1.33 µL T7 RNA Polymerase Mix, 5 µM DFHBI, 40 mM HEPES, 125 mM KCl, 5 mM $MgCl_2$, 1.25 µM folded Spinach Template, 62.5 mM NaCl, 25 nM mi141T7-Lock, and 0, 2.5, 5, 10, 25, 50, or 100 nM miRNA 141.*

Selectivity of miRNA detection and cross-talk determination were performed by assembling 4 identical master mixes according to the following protocol: To a 500 µL PCR tube, 100 µL NTP Buffer Mix, 20 µL T7 RNA Polymerase Mix, 30 µL DFHBI (50 µM, in DMSO), 30 µL 10x Spinach Buffer, 3.75 µL folded Spinach Template (100 µM), and 11.25 µL Nuclease-free water were added. The master mixes were briefly homogenized with a pipette. Afterward, 75 µL of the freshly snap-cooled mi21-, mi29a-, mi141- or mi155- T7-Lock (100 nM, in 250 mM NaCl) was added to each master mix, followed by a thorough homogenization with a pipette. Of each master mix, 18 µL was aliquoted into 15 wells of a 384-well plate. To three of those wells, 2 µL Nuclease-free water was added (negative controls), and in the remaining 12 wells, 2 µL of a 2.5 µM miRNA stock (miRNA21, miRNA29a, miRNA141, or miRNA155) was added in triplicate. The samples were overlaid with hexadecane and measured in the plate reader. *The final reaction volume of each sample was 20 µL and contained 6.67 µL NTP Buffer Mix, 1.33 µL T7 RNA Polymerase Mix, 5 µM DFHBI, 40 mM HEPES, 125 mM KCl, 5 mM $MgCl_2$, 1.25 µM folded Spinach Template, 62.5 mM NaCl, 25 nM T7-Lock (mi21-, mi29a-, mi141- or mi155- T7-Lock), and 250 nM miRNA (miRNA21, miRNA29a, miRNA141, or miRNA155), except for the negative control, where no miRNA was added.*

The obtained RFU values were plotted against time. The maximum RFU of the samples where the correct miRNA key was added, were compared to the blank as well as to samples with the false miRNA key at the same time point. The standard deviation between the triplicates and the ON/OFF ratio (RFU sample vs RFU blank) of each T7-Lock set were calculated with the following equations:

$$St.Dev.S = \sqrt{\frac{\sum(x_i - \bar{x})^2}{n-1}}$$

$$ON/OFF\ ratio = \frac{RFU\ Sample\ (at\ sample\ maximum)}{RFU\ Blank\ (at\ sample\ maximum)}$$

Selectivity of miDNA detection and cross-talk determination were performed in the exact manner as described in the section above (Selectivity of miRNA detection and cross-talk determination), with the only exception that miRNA keys were replaced with miDNA keys with the same nucleotide sequences and concentrations.

miRNA Signal Amplification Circuit

Site-specific transcription termination by Cx spacer modification was performed twice, once with a C3 spacer, and once with a C12 spacer in the X-Cx-Y-Template. For each experiment, two standard transcriptions with reaction volumes of 20 µL each were prepared. To a 200 µL PCR tube, 6.67 µL NTP Buffer Mix, 1.33 µL T7 Polymerase Mix, 2 µL 12.5 µM T7 promoter, 8 µL Nuclease-free water, and 2 µL 12.5 µM X-C12-Y-Template (Transcription A) or 2 µL 12.5 µM XY-template (Transcription B) were added. The samples were incubated at 37 °C for 3 hours.

The following samples were assembled in 200 µL PCR tubes; (A): 4 µL Nuclease-free water and 2 µL DY-647 labeled Y-Marker (5 µM), (B): 4 µL Nuclease-free water and 2 µL Atto-565 labeled X-Marker (5 µM), (C): 2 µL DY-647 labeled Y-Marker (5 µM), 2 µL Atto-565 labeled X-Marker (5 µM), and 2 µL X-Cx-Y-Template (1.25 µM), (D): 2 µL DY-647 labeled Y-Marker (5 µM), 2 µL Atto-565 labeled X-Marker (5 µM), and 2 µL XY-template (1.25 µM), (E): 2 µL DY-647 labeled Y-Marker (5 µM), 2 µL Atto-565 labeled X-Marker (5 µM), and 2 µL transcript A, (F): 2 µL DY-647 labeled Y-Marker (5 µM), 2 µL Atto-565 labeled X-Marker (5 µM), and 2 µL transcript B. All samples were incubated at room temperature for 3 hours. Afterward, the samples were 50% diluted in glycerol, of which 5 µL was pipetted in a 50 mL gel with 0.7 wt% Agarose, 1x TAE buffer, and 1.6x ROTI GelStain. The electrophoresis was run at room temperature for 45 minutes at 100 V with 1x TAE as a running buffer. After completion the gels were imaged on a INTAS *CHEMOSTAR Touch* fluorescence imager (INTAS Science Imaging), recording channels separately for ROTI, Atto 565, and DY-647 P1.

miRNA signal amplification with amplifier template was achieved by assembling a master mix with a volume of 270 µL. To a 500 µL PCR tube, 100 µL NTP Buffer Mix, 20 µL T7 RNA Polymerase Mix, 30 µL DFHBI (50 µM, in DMSO), 30 µL 10x Spinach Buffer, 3.75 µL folded Spinach Template (100 µM), 3 µL miRNA141 (10 µM), and 8.25 µL Nuclease-free water were added, followed by a thorough homogenization with a pipette. Afterward, 75 µL of a freshly snap-cooled mi141 T7-Lock (50 nM, in 250 mM NaCl) was added and the sample was again homogenized with a pipette. Of the master mix, 15 aliquots of 18 µL were made in a 384-well plate. To those wells either 2 µL 125 nM miR141-Amplification Template (C12-modified), 2 µL 125 nM miR141-Amplification Template (unmodified), or 2 µL Nuclease-free water was added in quintuplicates. The samples were overlaid with hexadecane and measured in the plate reader. *The final reaction volume of each sample was 20 µL and contained 6.67 µL NTP Buffer Mix, 1.33 µL T7 RNA Polymerase Mix, 5 µM DFHBI, 40 mM HEPES, 125 mM KCl, 5 mM $MgCl_2$, 1.25 µM folded Spinach Template, 62.5 mM NaCl, 12.5 nM mi141 T7-Lock, 100 nM miRNA, and 12.5 nM miR141-Amplification Template (C12-modified), 12.5 nM miR141-Amplification Template (unmodified), or no amplification template (negative control).*

miRNA Triggered Protein Expression

miRNA triggered mRNA transcription was achieved by first generating a DNA strand with a single stranded T7 promoter-binding region, followed by a ribosome binding sequence, and dsDNA strand containing the *mCherry* gene.

Generating sticky-end PCR products. The coding sequence of *mCherry* was extracted from the OSCA1.1 plasmid by means of PCR. The PCR samples were prepared in 20-fold each and consisted of 2 ng OSCA1.1 plasmid, 0.2 µM reverse primer (RP mCherry OSCA1.1), and 0.2 µM forward primer (A: FP-L-mCherry OSCA1.1, B: FP-L-C3-mCherry OSCA1.1, or C: FP-L-C12-mCherry OSCA1.1). To that, 0.2 mM dNTPs, 1x *Taq* buffer (buffer (10 mM Tris-HCl, 50 mM KCl, 1.5 mM $MgCl_2$) and 2.5 units *Taq* polymerase were added. The sample volume was adjusted to 100 µL by addition of Nuclease-free water. The PCR was performed on a qTower^3 Real-time Quantitative PCR (Analytik Jena) as follows: initial denaturation at 95 °C for 1 minute, followed by 30 cycles of denaturation at 95 °C for 30 seconds, annealing at 58 °C for 40 seconds and extension at 68 °C for 90 seconds. Followed by one final extension at 68 °C for 5 minutes after which all replicates were merged.

Agarose gel electrophoresis for confirmation sticky-end PCR product. 5 µL of the PCR samples (together with a negative control that did not contain the *Taq* polymerase) was mixed with a stochiometric amount of DNA strands Linker*-Promoter* and a DY-647P1-labelled T7 promoter and left to incubate at room temperature for 3 hours. Afterward, they were directly diluted by 50% using glycerol, and 8 µL of each sample was pipetted in a 50 mL gel with 0.7 wt% Agarose, 1x TAE buffer, and 1.6x ROTI GelStain. The electrophoresis ran at room temperature for 45 minutes at 100 V with 1x TAE as a running buffer. After completion the gels were imaged on a INTAS *CHEMOSTAR Touch* fluorescence imager (INTAS Science Imaging), recording channels separately for ROTI, and DY 647P1.

PCR product purification was performed by filtration with Amicon Ultracentrifugal filters (30 kDa cutoff, Merck Millipore), followed by a 500 µL TE buffer, and a double 500 µL Nuclease-free water washing step. The concentrations were determined with a NanoDrop 2000 C (Fisher Scientific) and diluted to 3500 ng/µL with Nuclease-free water.

T4 ligation of T7 promoter binding site to PCR products. was performed in triplicate. In separate 200 µL PCR tubes, 10 µL 10x T4 ligase buffer (500 mM Tris-HCl, 100 mM MgCl2, 10 mM ATP, 100 mM DTT, pH 7.5 @25 °C), 10 µL T4 Ligase (400,000 units/mL), 10 µL Linker*-Promoter* (0.8 µM, containing a T7-binding site, and ribosome binding sequence), 10 µL of PCR product A, B or C (3500 ng/ul; 0.8 µM each), and 60 µL Nuclease-free water were added. The samples were incubated overnight in an Eppendorf Thermomixer at 16 °C. Thereafter, replicates were merged and ligated templates were purified and quantified in the same manner as described for the PCR product purification. The concentrations were diluted to 1750 ng/µL with Nuclease-free water.

T7 promoter-triggered protein expression from non-, C3-, and C12-modified templates
was performed by assembling a master mix with a volume of 162 µL. From the PURExpress TX-TL kit, 37.5 µL Solution A, and 28.1 µL Solution B are added to a 200 µL PCR tube. Next, 1.9 µL Murine RNase inhibitor (40,000 units/mL), 18 µL T7 promoter DNA (12.5 µM), and 76.5 µL Nuclease-free water were added. The master mix was homogenized and aliquoted (9x18 µL) into a 384-well plate. To those wells, 2 µL of DNA templates A (unmodified primer), B (C3-modified primer) or C (C12-modified primer) (1750 ng/µL each), were added in triplicates. The samples were overlaid with hexadecane and measured in the plate reader. *The final reaction volume of each sample was 20 µL and contained 4.2 µL solution A, 3.1 µL Solution B, 8.4 units Murine RNase inhibitor, 1.25 µM T7 promoter DNA, and 175 ng/µL of DNA templates A, B or C (200 nM).*

miRNA-triggered transcription and translation visualization under a microscope was performed using a Teflon mold shaped like a cherry (fabricated by CNC milling (CNC-Portal-milling machine (BZT PF 750-P)). There were two separated units, with combined dimensions of 20 x 8 mm. For the translation module, a 192 µL reaction volume was used. To a 200 µL PCR tube, 40 µL Solution A and 30 µL Solution B were added from the PURExpress TX-TL kit. Next, 2 µL Murine RNase inhibitor (40,000 units/mL), 20 µL DNA template B (1750 ng/µL, generated from the C3-modified primer) and 70.4 µL Nuclease-free water added. After a fast homogenization by pipette, 24 µL of a freshly snap-cooled mi141 T7-Lock (100 nM, in 250 mM NaCl) was added, followed by a quick homogenization, and addition of 4.6 µL miRNA 141 (10 µM). *The final sample contained 412 units/mL RNase inhibitor, 175 ng/ µL DNA template B (200 nM), 12.5 nM mi141 T7-Lock, and 250 nM miRNA 141.* The sample was quickly transferred into the body of the fruit in the Teflon mold. For the transcription module, a 160 µL reaction volume was used. to a 200 µL PCR tube, 53.4 µL NTP Buffer Mix, 10.6 µL T7 RNA Polymerase Mix, 16 µL DFHBI (50 µM, in DMSO), 16 µL 10x Spinach Buffer, 2 µL folded Spinach Template (100 µM), and 18 µL Nuclease-free water were added. The master mixes were briefly homogenized with a pipette. Afterward, 40 µL of the freshly snap-cooled mi141 T7-Lock (50 nM, in 250 mM NaCl), and 4 µL miRNA 141 (10 µM) were added, followed by a thorough homogenization with a pipette. *The final sample contained 5 µM DFHBI, 40 mM HEPES, 125 mM KCl, 5 mM $MgCl_2$, 1.25 µM folded Spinach Template, 62.5 mM NaCl, 12.5 nM mi141 T7-Lock and 250 nM miRNA 141.* The sample was quickly transferred into the leaf of the fruit in the Teflon mold. The samples were covered with an EASYseal sealing film (Greiner) and placed on a 37 °C heating stage for the duration of the experiment. Every minute a fluorescent image was obtained with a Nikon SMZ25 fluorescence microscope with 470 nm LED irradiation (Spinach aptamer) and 555 nm (mCherry protein).

miRNA-triggered transcription vs. translation was compared by first assembling a master mix for the transcription module with a volume of 160 µL total. To a 200 µL PCR tube, 43.4 µL NTP Buffer Mix, 10.6 µL T7 RNA Polymerase Mix, 16 µL DFHBI (50 µM, in DMSO), 16 µL 10x Spinach Buffer, 2 µL folded Spinach Template (100 µM), and 6 µL Nuclease-free water were added. The master mix was briefly homogenized with a pipette. Afterward, 40 µL of a freshly snap-cooled mi141 T7-Lock (50 nM, in 250 mM NaCl) was added The master mix was homogenized and aliquoted (8x18 µL) into a 384-well plate. To those aliquots either 2 µL Nuclease-free water (negative control), or 2 µL of a miRNA 141 stock (2.5 µM) was added. *The final reaction volume of each sample was 20 µL and contained 6.67 µL NTP Buffer Mix, 1.33 µL T7 RNA Polymerase Mix, 5 µM DFHBI, 40 mM HEPES, 125 mM KCl, 5 mM $MgCl_2$, 1.25 µM folded Spinach Template, 62.5 mM NaCl, 12.5 nM mi141T7-Lock, and 0 or 250 nM miRNA 141.* For the translation module, to a 200 µL PCR tube, 33.4 µL Solution A and 25 µL Solution B were added from the PURExpress TX-TL kit. Next, 1.7 µL Murine RNase inhibitor (40,000 units/mL), 16.7 µL DNA template B (1750 ng/µL, generated from the C3-modified primer) and 59.4 µL Nuclease-free water added. After a fast homogenization by pipette, 20 µL of a freshly snap-cooled mi141 T7-Lock (100 nM, in 250 mM NaCl) was added, followed by a thorough homogenization by pipette. Of the master mix, 18 µL was aliquoted into 8 wells of a 384-well plate. To those wells either 2 µL Nuclease-free water (negative control), or 2 µL of a miRNA 141 stock (2.5 µM) was added. *The final sample contained 412 units/mL RNase inhibitor, 175 ng/ µL DNA template B (200 nM), 12.5 nM mi141 T7-Lock, and 0 or 250 nM miRNA 141.* The samples were overlaid with hexadecane and measured on the plate reader.

4. Supplementary Table

Table S1: Oligonucleotide sequences as purchased from Biomers, with their name, sequence, purification method and modifications. The T7 promoter regions are underlined, and the lock regions are shown in bold.

Name	Sequence (5’→ 3’)	Purification method	Modification
**T7 Promoter (DNA)**	CGC TAA TAC GAC TCA CTA TA	Cartridge	none
**T7 Promoter (RNA)**	CGC UAA UAC GAC UCA CUA UA	HPLC	none
**Spinach Template (folded)**	GGA GCT CAC ACT CTA CTC AAC AGT AGC<br>GAA CTA CTG GAC CCG TCC TTC ACC CTA TAG<br>TGA GTC GTA TTA GCG AGT ATA GGG	HPLC	none
**Spinach Template (linear)**	GGA GCT CAC ACT CTA CTC AAC AGT AGC<br>GAA CTA CTG GAC CCG TCC TTC ACC CTA TAG<br>TGA GTC GTA TTA	HPLC	none
**miDNA 21**	TAG CTT ATC AGA CTG ATG TTG A	HPLC	none
**miRNA 21**	UAG CUU AUC AGA CUG AUG UUG A	HPLC	none
**mi21-T7 Lock**	**TCA ACA TCA GTC TGA TAA GCT A**TT TTT TTT<br>TAG CTT ATC ATA ATA CGA CTC GAC TGA TGT	HPLC	none
**miDNA 29a**	TAG CAC CAT C TGA AAT CGG TTA	HPLC	none
**miRNA 29**	UAG CAC CAU CUG AAA UCG GUU A	HPLC	none
**mi29a-T7 Lock**	**TAA CCG ATT TCA GAT GGT GCT A**TT TTT TTT<br>TAG CAC CAT C TAA TAC GAC TCT GAA ATC<br>GG	HPLC	none
**miDNA 155**	TTA ATG CTA A TC GTG ATA GGG GTT	HPLC	none
**miRNA 155**	UUA AUG CUA AUC GUG AUA GGG GUU	HPLC	none
**mi155-T7 Lock**	**AAC CCC TAT CAC GAT TAG CAT TAA** TTT TTT<br>TTA ATG CTA A TCT AAT ACG ACT CGT GAT<br>AGG G	HPLC	none
**miDNA 141**	TAA CAC TGT C TGG TAA AGA TGG	HPLC	none
**miRNA 141**	UAA CAC UGU CUG GUA AAG AUG G	HPLC	none
**mi141-T7 Lock**	**CCA TCT TTA CCA GAC AGT GTT A**TT TTT TTT<br>TAA CAC TGT C TAA TAC GAC TC TGG TAA AGA	HPLC	none
**miR141-Amplification Template (unmodified)**	TAA CAC TGT C TGG TAA AGA TGG TTT TTC<br>CAT CTT TAC CAG ACA GTG TTA CCC TAT AGT<br>GAG TCG TAT TAG CGA GTA TAG GG	HPLC	none
**miR141-Amplification Template (C12-modified)**	TAA CAC TGT C TGG TAA AGA TGG TTT TT1<br>CCA TCT TTA CCA GAC AGT GTT ACC CTA TAG<br>TGA GTC GTA TTA GCG AGT ATA GGG	HPLC	Internal C12 spacer (at '1' position)
**XY-Template**	A CTA CAG CCG CAC AAG AAA CCC AGA ACA<br>TCA GAC CAA CAC AAG AAA CCC AAC CCA<br>GAA CAT CAT TGC TCC TCT TAC GTC ATT ATT<br>CAT CAG TAC TAC CCG TCC TTC ACC CTA TAG<br>TGA GTC GTA TTA GCG AGT ATA GGG	HPLC	
**X-C12-Y-Template**	A CTA CAG CCG CAC AAG AAA CCC AGA ACA<br>TCA GAC CAA CAC AAG AAA CCC AAC CCA<br>GAA CAT CAT TGC TCC TCT TAC GTC ATT ATT<br>CAT CA1G TAC TAC CCG TCC TTC ACC CTA<br>TAG TGA GTC GTA TTA GCG AGT ATA GGG	HPLC	Internal C12 spacer (at '1' position)

**X-C3-Y-Template**	A CTA CAG CCG CAC AAG AAA CCC AGA ACA TCA GAC CAA CAC AAG AAA CCC AAC CCA GAA CAT CAT TGC TCC TCT TAC GTC ATT ATT CAT CA1G TAC TAC CCG TCC TTC ACC CTA TAG TGA GTC GTA TTA GCG AGT ATA GGG	HPLC	Internal C3 spacer (at '1' position)
**X-Marker**	ACT ACC CGT CCT TCA	HPLC	5’ Atto 565
**Y-Marker**	AGA ACA TCA GAC CAA	HPLC	5’DY-647P1
**FP-L-mCherry OSCA1.1**	GCA ATG ATA AAC GAG CAC GAT GAT AAT ATG GCC ACA ACC ATG G	Cartridge	none
**FP-L-C3-mCherry OSCA1.1**	GCA ATG ATA AAC GAG 1CA CGA TGA TAA TAT GGC CAC AAC CAT GG	HPLC	Internal C3 spacer (at '1' position)
**FP-L-C12- mCherry OSCA1.1**	GCA ATG ATA AAC GAG 1CA CGA TGA TAA TAT GGC CAC AAC CAT GG	HPLC	Internal C12 spacer (at '1' position)
**RP mCherry OSCA1.1**	ATC TAG AGT CGC GGC CGC TTA CTT	Cartridge	none
**Linker*-Promoter***	CTC GTT TAT CAT TGC ATA TCT CCT TCT GTT TCT CCC TAT AGT GAG TCG TAT TAG CGA GTA TAG GG	HPLC	5' phosphate
**T7 Promoter (dye labelled)**	CGC TAA TAC GAC TCA CTA TA	HPLC	5’DY-647P1

5. Supplementary Figures

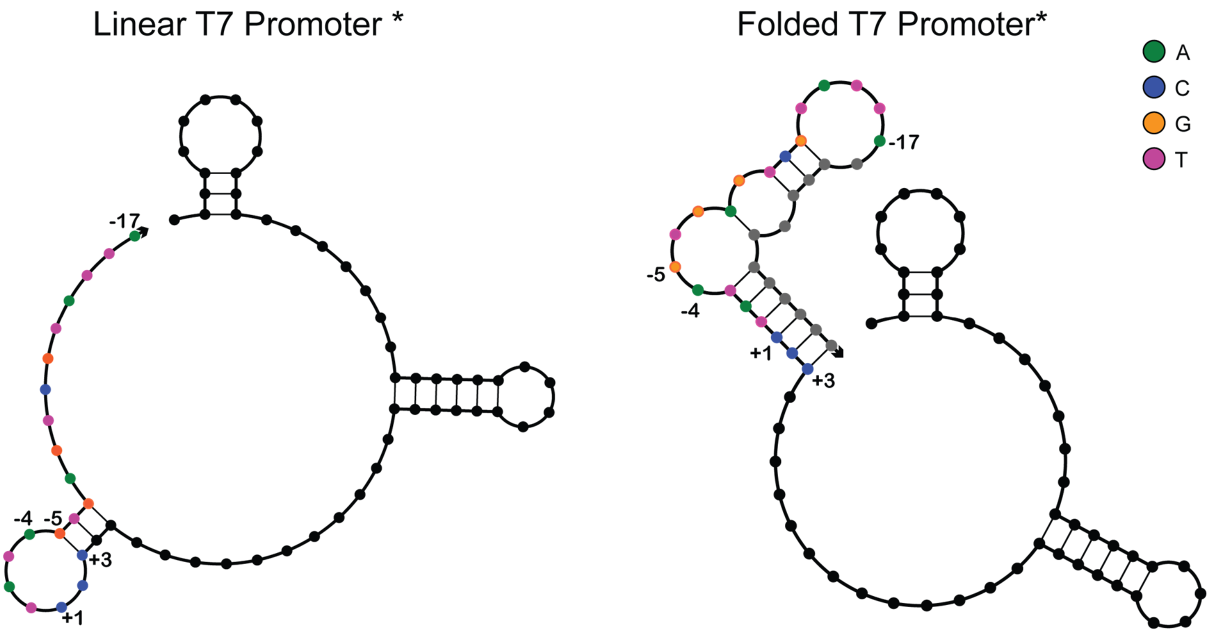

Figure S1. Secondary structure linear template vs folded template. Comparison of the minimum free energy proxy secondary structures of a linear T7 template, and a folded T7 template encoding the Spinach aptamer. The nucleotides in the promoter sequences are illustrated in color, whilst the sequences corresponding to the Spinach aptamer are represented in black. The nucleotides that cause the secondary structure in the folded T7 Promoter*, are illustrated in grey. Here, the melting region (-4 → +3) is mainly double stranded, aiding the binding region (-17 → -5) to be shaped in a complex secondary structure. The MFE proxy structures were predicted by NUPACK at temperature settings of 37 °C, 125 mM NaCl and 6 mM $MgCl_2$.

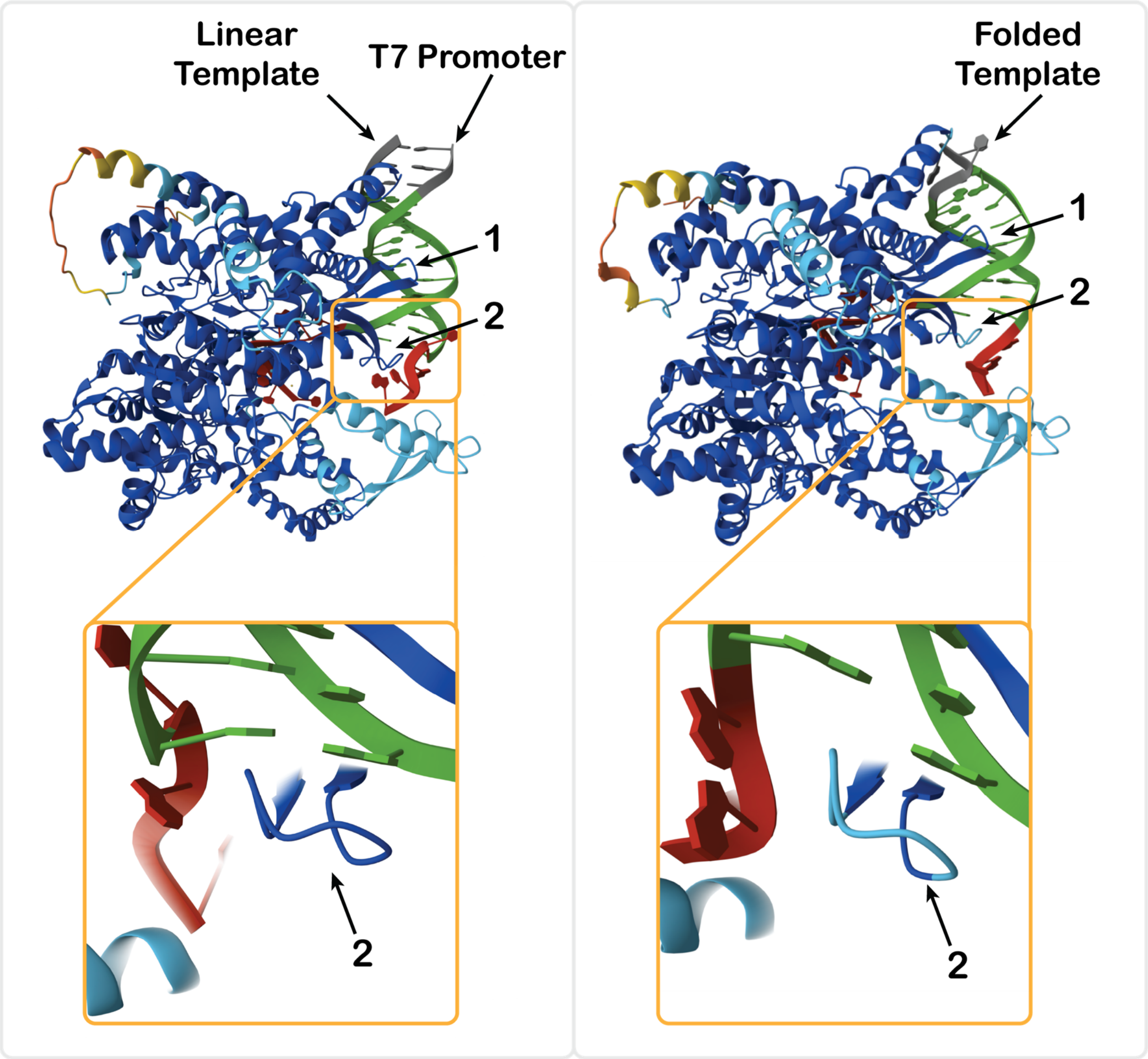

Figure S2. Interactions of T7 RNAP with folded template vs linear template + T7 promoter by Alphafold 3. AlphaFold simulation of T7 RNAP interaction with folded template vs linear template + T7 promoter. **Left:** T7 RNAP enzyme interacting with a linear DNA template and T7 promoter. The Beta Hairpin (1) recognizes the duplex in the binding region (green), and positions the template on the active site of the enzyme. In the melting region (red) the intercalating loop (2) interacts with the -4 position of the T7 promoter, opening the transcription bubble, and thus initiating transcription.[1,2] **Right:** T7 RNAP interaction with the folded template and no T7 promoter. The Beta Hairpin (1) fully recognizes the template in the binding region (green), as it would recognize a linear template + T7 promoter. This places the template in the active site of the enzyme. Due to sequence variations downstream of the folded template, the interactions with the intercalating loop (2) in the melting region (red) are severely disturbed. This presumably prevents the intercalating loop from introducing the template to the transcription bubble.

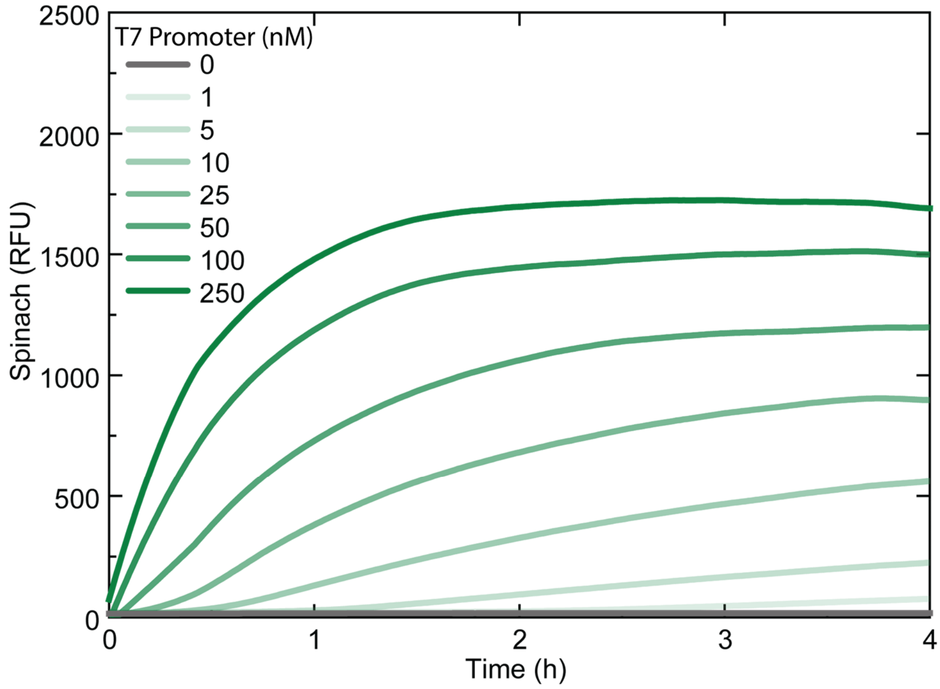

Figure S3. Dependency of T7 promoter concentration on quantity RNA transcribed. Real-time fluorescence measurement of T7 promoter-triggered Spinach transcription for varying concentrations of T7 promoter. Conditions: HiScribe T7 Quick High Yield RNA Synthesis Kit (NEB) according to manufacturer's conditions, 1.25 µM folded Spinach template, 40 mM HEPES, 125 mM KCl, 5 mM MgCl2, 62.5 mM NaCl, and 5 µM DFHBI at 37 °C. Input: 0, 1, 5, 10, 25, 50, 100 and 250 nM T7 promoter. Representative single measurements shown.

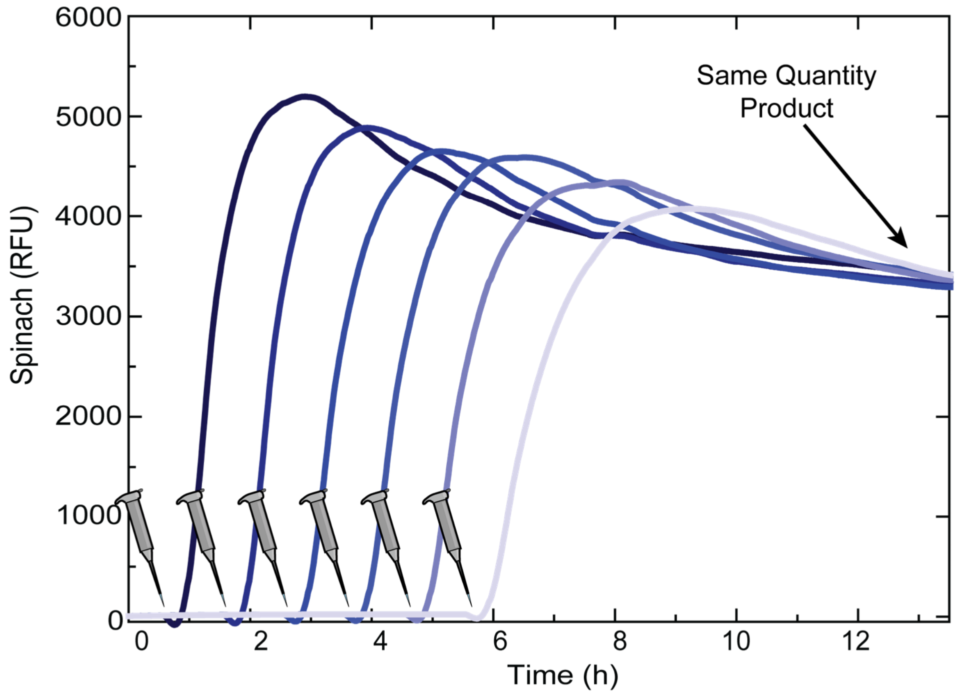

Figure S4. Transcriptional activity following prolonged dormancy periods. Real-time fluorescence measurement of dormant Spinach transcription samples which are activated at different points in time by the delayed addition of the T7 promoter in presence of a folded T7 template. The transcription system can remain dormant for at least 6 hours, with a maintained possibility for activation. The transcriptional activity decreases slightly with increased dormancy time, which can be observed as a reduced steepness of the slope during the transcriptional rise. This phenomenon is associated with the 'overshoots' observed as peak maxima. In this phase the templates are presumably still inhibited by the T7 RNAP and incomplete transcripts allowing free Spinach RNA to bind to the DFHBI and increase fluorescence. Once transcriptional activity decreases, the template binds with some of the RNA product, causing a reduction in RNA-DFHBI complexes, and thus stabilization of the fluorescence level. This results in varying curve maxima and transcription rates over prolonged dormancy times, but stable final product quantities. Conditions: HiScribe T7 Quick High Yield RNA Synthesis Kit (NEB) according to manufacturer's conditions, 1.25 µM folded Spinach template, 40 mM HEPES, 125 mM KCl, 5 mM $MgCl_2$, and 5 µM DFHBI at 37 °C. Input: 1.25 µM T7 promoter, added at *t* = 0, 1, 2, 3, 4, 5 and 6 hours. Representative single measurements shown

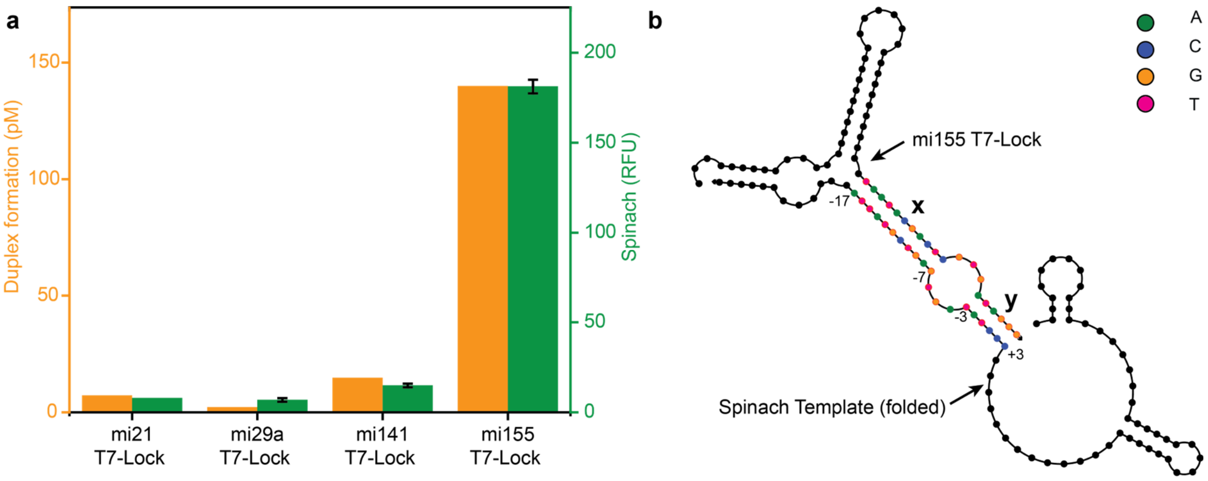

Figure S5. Hybridization between T7-Locks and Spinach template in absence of miRNA keys as calculated by NUPACK. (a) Comparison of concentrations of unsolicited duplexes formed between the folded Spinach template and the mi21, mi29a, mi141 and mi155 T7-Locks (orange) and the experimentally obtained Spinach fluorescence (RFU) values (green) corresponding to the blank reactions shown in Figure 3e of the main text. The somewhat higher binding of miRNA155 Lock with the template explains the lower ON/OFF ratio (see Figure 3e). (b) Minimum free-energy (MFE) proxy structure of the unsolicited mi155 T7-Lock/folded Spinach template hybrid predicted by NUPACK. The *x* region of the mi155 T7-Lock, present in all T7-Locks, activates transcription by hybridizing to the -17 → -7 region of the T7 promoter binding site. The *y* region, specific to miRNA155, coincidentally complements the -3 → +3 region of the T7 promoter binding site. This additional complementarity lowers the hybridization energy between the mi155 T7-Lock and the folded Spinach template, slightly shifting the equilibrium towards an activated template. NUPACK simulations were performed at 37 °C, 125 mM NaCl, 6 mM $MgCl_2$, 25 nM mi21, mi29a, mi141 or mi155 T7-Locks and 1.25 µM folded Spinach template. Experimental 'blank' conditions: HiScribe T7 Quick High Yield RNA Synthesis Kit, 1.25 µM folded Spinach template, 25 nM miX-T7 Lock, 40 mM HEPES, 125 mM KCl, 5 mM $MgCl_2$, 62.5 mM NaCl, and 5 µM DFHBI at 37 °C. Input: 0 nM miRNA X (blank). Data are averages of n = 5 and error bars as standard deviations, values taken at peak maxima from Figure 3e.

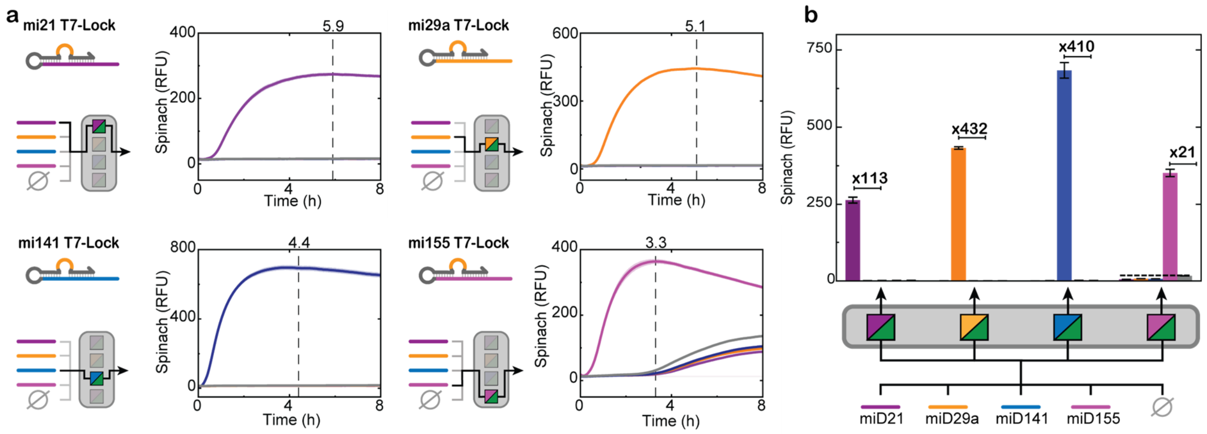

Figure S6. Selective detection of miDNA input. (a) Selective detection of miDNA input, using a mi21 T7-Lock, mi29a T7-Lock, mi141 T7-Lock, or a mi155 T7-Lock. To each T7-Lock, miDNA-21, 29a, 141 and 155 were added separately, after which transcription of the Spinach aptamer was recorded. Fluorescent signals above blank level were only observed when the corresponding miDNA input was added. Conditions: HiScribe T7 Quick High Yield RNA Synthesis Kit (NEB) according to manufacturer's conditions, 1.25 μM folded Spinach template, 25 nM miX-T7 Lock, 40 mM HEPES, 125 mM KCl, 5 mM $MgCl_2$, 62.5 mM NaCl, and 5 μM DFHBI at 37 °C. Input: 250 nM miRNA X, or 0 nM miRNA X (blank). All curves are averages of n = 5 measurements. Shaded areas (partly invisible as too small) represent the standard deviation. (b) Overview of the output RFU maxima from each T7-Lock along with their respective miRNA key obtained from the curves in panel a. Along with signals obtained the same T7-Lock channels, but different miRNA inputs, at the same time points. The bar chart shows the average of 5 curves along with the corresponding standard deviation.

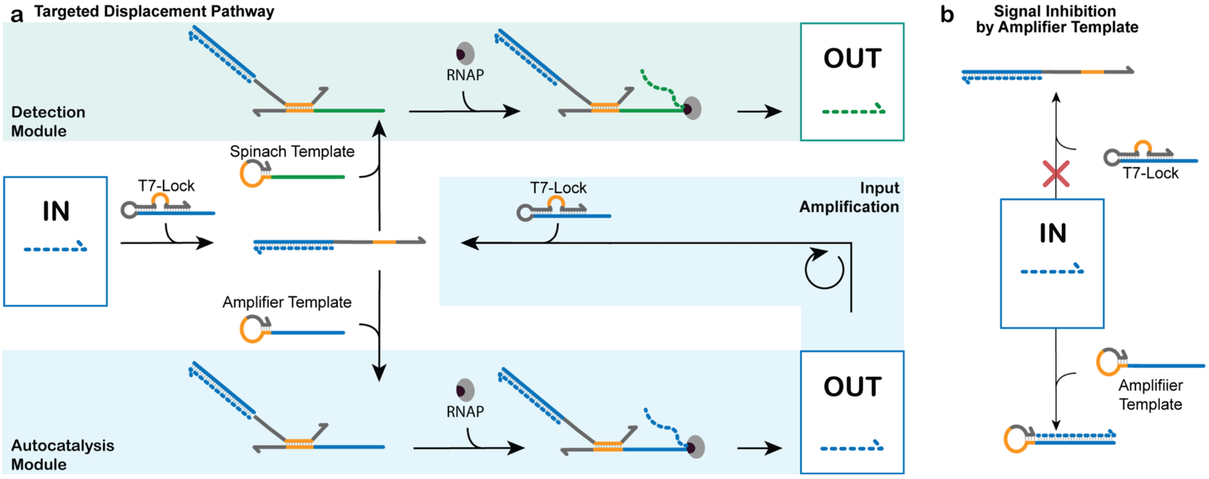

Figure S7. Limitations of internal signal amplification template. (a) Envisioned pathway where miRNA as a trigger opens the T7-lock, thus exposing the T7-promoter. Once the promoter is exposed, the T7 promoter can interact with the spinach template to produce a fluorescent signal. Next to that, a template is present that encodes the transcription of the input signal itself, which would cause internal positive feedback. **However, (b) shows that instead of opening the T7-lock, the miRNA will rather hybridize with the amplification template itself, as it is thermodynamically less hindered.** This will cause signal inhibition and thus eliminate the possibility for T7-lock activated transcription and therefore also signal amplification.

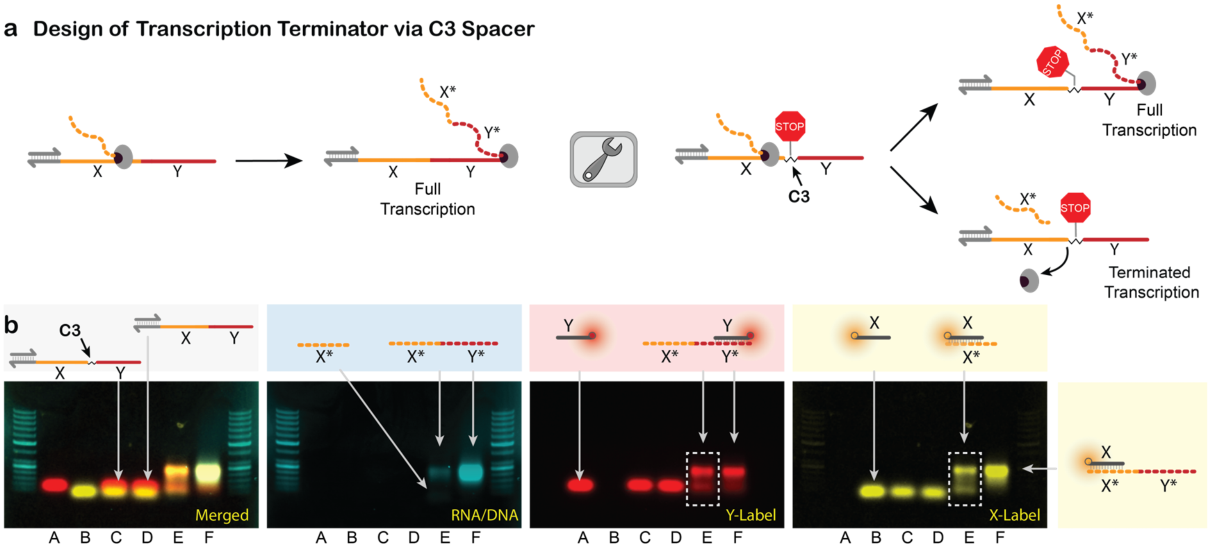

Figure S8. Limited transcription termination with C3 modification in template. (a) Schematic representation of limited site-specific transcription termination caused by a C3 spacer modification in the template. (b) AGE: Fluorescence images of RNA products, templates and probe strands to characterize transcribed products with or without a C3-modified template. (A): DY-647 labeled Y-Marker, (B): Atto-565 labeled X-marker, (C): X-Marker, Y-Marker, and C3-modified template, (D): X-Marker, Y-Marker, and unmodified template, (E): X-Marker, Y-Marker, and RNA transcribed from C3-modified template, (F): X-Marker, Y-Marker, and RNA transcribed from unmodified template. 50 bp DNA ladders as reference.

Note: The Y-Marker (red channel; important band highlighted by rectangle) interacts with the RNA transcribed from the C3-modified template (lane E); rather similarly as for the non-modified template (F). This confirms the presence of RNA with the full X*-Y* sequence, indicating inefficient termination at the C3-modification site. The X-Marker (yellow channel; important bands highlighted by rectangle) in well E shows 2 bands. This indicates that both X* and X*Y* RNA are present, indicating that some termination occurs, but at much lower levels as with the C12 modifier presented in Figure 4b in the main manuscript.

6. References

(1) Tahirov, T. H.; Temiakov, D.; Anikin, M.; Patlan, V.; McAllister, W. T.; Vassylyev, D. G.; Yokoyama, S. Structure of a T7 RNA Polymerase Elongation Complex at 2.9 A˚ Resolution. *Nature* **2002**, *420*, 43–50. https://doi.org/10.1038/nature01129.

(2) Yin, Y. W.; Steitz, T. A. Structural Basis for the Transition from Initiation to Elongation Transcription in T7 RNA Polymerase. *Science* **2002**, *298*, 1387–1395. https://doi.org/10.1126/science.1077464.
